# Tenth International Symposium on Intensive Care and Emergency Medicine for Latin America: São Paulo, Brazil. 9 - 13 July, 2019

**DOI:** 10.1186/s13054-019-2503-9

**Published:** 2019-07-08

## Basic Science

### P01 KIR genes *2DS1*, *3DS1*, and *2DL5* are associated with lower organ dysfunction in a cohort of critical care patients without sepsis

#### Luciana Mello de Oliveira^1^, Jaqueline Beppler^1^, Juliana Lindenau^1^, Pamela Portela^2^, Fernando Suparregui Dias^3^, Clarice Sampaio Alho^4^, Felipe Dal-Pizzol^5^, Luiz Fernando Jobim^2^, Mariana Jobim^2^, Rafael Roesler^1^

##### ^1^UFRGS – Universidade Federal do Rio Grande do Sul, Porto Alegre, Rio Grande do Sul, Brazil; ^2^HCPA – Porto Alegre Clinical Hospital, Porto Alegre, Rio Grande do Sul, Brazil; ^3^HP – Pompéia Hospital, Caxias do Sul, Rio Grande do Sul, Brazil; ^4^PUCRS –Pontifical Catholic University of Rio Grande do Sul, Porto Alegre, Rio Grande do Sul, Brazil; ^5^SJH – São José Hospital, Criciúma, Santa Catarina, Brazil

**Background**

Natural killer cells are part of the innate immune system and their activity is regulated by activating and inhibitory signals transduced by killer cell immunoglobulin-like receptors (KIR). Seventeen KIR receptors have been characterized in humans: eight inhibitory types (2DL1–3, 2DL5A and B, and 3DL1–3), seven activating types (2DS1–5, 3DS1, and 2DL4), and two pseudogenes that do not encode a functional KIR receptor. We previously reported a reduced frequency of activating genes in patients with sepsis [1].

**Objective**

We aimed to evaluate KIR genes in 60 critically ill patient without sepsis and their association with each system or organ dysfunction represented by the Sequential Organ Failure Assessment (SOFA) score.

**Methods**

Subjects were southern Brazilian residents, with a majority of subjects having European ethnicity and a minor number of individuals with African genetic traits, with an age range from 14 to 94 years admitted to the Intensive Care Unit, São Lucas Hospital, Pontifical Catholic University of Rio Grande do Sul, in Porto Alegre, Brazil, between 2004 and 2010. DNA samples of 60 critical care patients were genotyped using the polymerase chain reaction method with sequence-specific oligonucleotide (PCR-SSO kit, One Lambda® Inc, Woodland Hills, USA) for 16 KIR genes. SOFA scores were collected at admission and every day during the intensive care unit (ICU) stay. To evaluate the influence of individual KIR genes on each of the six system or organ dysfunctions as evaluated by the SOFA score, Poisson regression analysis was performed. A *p* value ≤0.05 after correction was considered to indicate a statistically significant difference.

**Results**

The number of organs and systems in dysfunction were 3.87 ± 1.46 in the population: 4.07 ± 1.36 for septic patients and 3.17 ± 1.40 for controls. The KIR genes *2DL5*, *2DS1*, and *3DS1* were associated with not presenting cardiac dysfunction at any moment during the ICU stay (IC 0.76 (0.60–0.95), *p* = 0.008; IC 0.73 (0.58–0.94), *p* = 0.015; IC 0.71 (0.55–0.91), *p* = 0.014, respectively). The KIR gene *2DL5* was associated with not presenting renal dysfunction at any moment during the ICU stay (IC 0.78 (0.62–0.98), *p* = 0.037).

**Conclusions**

Although the number of patients is small, this preliminary study shows that the expression of KIR genes *2DS1* and *3DS1* may protect against cardiac dysfunction, and KIR gene *2DL5* may protect against cardiac and renal dysfunction in critical care patients. Biological mechanisms explaining why presenting certain KIR genes or haplotypes influence organ dysfunction are not yet known; perhaps expressing a greater number of activating KIR genes results in a more effective response against critical disease. Further studies are needed to translate this genetic observation into clinical evidence.

**Acknowledgments**

This study was approved by the Research Ethics Board of the Porto Alegre Clinical Hospital and was conducted in accordance with the Declaration of Helsinki. All subjects or their surrogates received detailed explanations and provided written consent prior to inclusion.

**Reference**

[1] Oliveira LM, Portela P, Merzoni J, et al. Reduced frequency of two activating KIR genes in patients with sepsis. Hum Immunol 2017;78:363–9.

## Cardiology

### P02 Use of mechanical circulatory support in the Brazilian public health system: a distant reality

#### Elaine Júlian da Fonseca^1^, Ana Cláudia Costa^1^, Nathália Lima^1^, Débora Braga^1^, Ana Couto^1^, Pedro Esaki^1^, Gabriela Amaral^1^, Paula Tolentino^1^, Giuliano Generoso^2^

##### ^1^UNICEPLAC – Centro Universitário do Planalto Central Apparecido Santos. Gama, DF, Brazil; ^2^HSL – Cardiology Center, Hospital Sírio Libanes, São Paulo, SP, Brazil

**Background**

Heart failure is a clinical entity with a high prevalence, mortality rate, and rehospitalization rate. Mechanical circulatory support devices represent a breakthrough in the therapeutic approach and became a reality after they demonstrated good results in developed countries.

**Objective**

We aimed to evaluate the use of circulatory assist devices offered by the Brazilian public health system.

**Methods**

This is a cross-sectional, retrospective, observational study of data collected through the hospital information system of the public Health system (SIH/DataSUS) from January 2008 to December 2017. The data from 2018 were excluded from the sample due to the possibility of updates.

**Results**

The total use of circulatory assist devices in the evaluated period was 346 with an increase from 2014; the annual average was of 57 ± 11.16. Of the total, 76% (*n* = 263) were requested as an emergency, with the remainder were installed as elective surgery. Only 13 of 27 federative units (48.14%) performed this procedure in the last decade, with no records of their use in the north of Brazil. Less than half (46.15%) of those federative units that used the resource used it five times or less. The southeast has the highest incidence (*n* = 217, 62.72%) followed by the midwest (*n* = 54, 15.61%) with an emphasis on São Paulo (*n* = 185, 53.47%) and the Federal District (*n* = 52, 15.03%). The total cost of hospitalization per procedure showed great variation, extending from R$266.18 to R$37,902.18, with an average of R$5,500.00. The mortality rate was 35 ± 14.23, which was higher in the south (58%) and lower in the midwest (25.9%). There are no recorded data that differentiate the types of circulatory assistance provided in the public health system, nor is the INTERMACS or equivalent score available.

**Conclusions**

The amount of mechanical circulatory support used in the last decade is a small significative amount, with none registered in more than half of the country. The available data do not differentiate the type of assistance provided, nor the profile of the patients in cardiogenic shock that would benefit from the therapy, as recommended by the Guidelines for Mechanical Circulatory Support of the Brazilian Society of Cardiology since 2016. Therefore, it reinforces the necessity to create a national registry to enable the analysis, comparison, and evolution of the use of devices in the country, such as the successful registries in Europe (EUROMACS), the United States (INTERMACS), and internationally (IMACS).

### P03 Angiographic aspects and profile of young patients (less than 40 years old) with acute myocardial infarction treated in hospitals in the state of São Paulo

#### Lucas S Macedo^1^, Rodrigo Balada^1^, Pedro GMB Silva^2^

##### ^1^CUSC – Centro Universitário São Camilo, São Paulo, SP, Brazil; ^2^TotalCor – Hospital TotalCor, São Paulo, SP, Brazil

**Background**

Acute myocardial infarction (AMI) is an unexpected event in individuals under the age of 40 years. It is important to analyze these patients in order to draw a profile of them and identify a possible mechanism in common for such an unexpected event.

**Objective**

The present study aimed to describe the physiopathology of young people under 40 years of age who suffered an AMI by correlating the types of infarction, associated mechanism, presence of comorbidities, and mortality rates.

**Methods**

Retrospective analysis was performed on the myocardial infarction database of a private network of hospitals and, among 2783 cases of AMI attended in 15 hospitals in the state of São Paulo in the period between January 2014 and December 2018, 62 patients were <40 years old (2.15%). The cases were separated according to the ECG, whether or not there was cardiac catheterization, angiographic aspects and the type of infarction, and the probable mechanism of myocardial infarction in cases of AMI type 2.

**Results**

Of the patients analyzed, 53 (85.4%) had atherothrombotic disease (AMI type 1) as a mechanism while (14.5%) were patients with type 2 AMI. The mechanisms responsible for type 2 AMI were most commonly coronary embolism (44%), spontaneous coronary dissection (33%), and imbalanced supply × demand (22%). Diagnosis of ST-elevation myocardial infarction (STEMI) represented 47% of the type 1 AMI and 11% of the type 2 AMI. Among the AMI type 1 with atherothrombotic disease, 50% had lesions in the left anterior descending artery (LAD) as the most important mechanism revealed by catheterization and, of these, 35% had lesions present in the proximal third. It was also observed that 25% of the cases had lesions in the right coronary artery (RCA) as the most important mechanism and, among these cases, 50% presented with lesions in the middle third of the artery. About 20% of the patients who had AMI type 2 presented with hypertrophy of the left ventricle and this finding related to the event of imbalance of supply × demand). It was also found that, among the patients with AMI <40 years, 40% were smokers (41% of AMI type 1 and 37.5% of AMI type 2).

**Conclusions**

More than 85% of AMI cases in patients <40 years old is by atherothrombotic disease and of these in almost half shows STsegment elevation. The most frequente cause of infarction type 2 was the coronary embolism and smoking was very prevalent risk factor.

### P04 Clinical presentation and long-term evolution in acute myocarditis: data from the multicenter registry ROAD-Brazil

#### Pedro de Barros e Silva^1^, Alexandre Soeiro^2^, Thiago Andrade Macedo^1,2^, Aline Siqueira Bossa^2^, Luciana Baptista^1^, Valter Furlan^1^, Mucio Tavares^2^

##### ^1^Totalcor – Hospital Totalcor, Sao Paulo, SP, Brazil; ^2^Incor – Instituto do Coração HC FMUSP, Sao Paulo, SP, Brazil

**Background**

Myocarditis is a generic term that encompasses a broad group of clinical situations that present with inflammation of the myocardium. A frequent but not very well explored group is myocarditis that mimics a picture of acute myocardial infarction.

Objective

We aimed to evaluate the clinical and laboratory pattern in the short and long term in a cohort of patients with acute myocarditis diagnosed after exclusion of coronary disease.

**Methods**

This was a multicenter observational study that included all consecutive patients presenting with chest pain and positive troponin without any significant obstruction on coronary angiography (invasive or noninvasive) in a 3-year period with a diagnosis of acute myocarditis based on characteristic late enhancement in the complementary investigation. The clinical and laboratory characteristics were analyzed at the baseline and the outcomes were evaluated every 6 months after discharge.

**Results**

A total of 61 cases of myocarditis were diagnosed in this period (54.1% presented a diagnosis of associated pericarditis) and all cases were considered to be of viral etiology, although 14.7% of the patients had no infectious disease 30× the limit of normality. The levels of these markers showed variations between measures >30%, but the serial curve already started in the descending phase in 81.9% of the cases. The clinical and laboratory characteristics are presented in Table 1. Only one patient died due to cardiogenic shock (in-hospital mortality of 1.6%, 95% confidence interval (CI) 0.01–9.5%). At a median of 2.5 years there was no death after hospital discharge and there was only one re-hospitalization (for heart failure).

**Conclusions**

More than half of the cases of acute myocarditis presented altered electrocardiogram (EKG) with an associated diagnosis of pericarditis and more than 20% presented segmental alterations to the echocardiogram. The prognosis was good in the long term, although 10% remained with left ventricular dysfunction. The categorization of myocarditis in more homogenous groups will allow a better understanding of the natural history and the generation of better hypotheses of potentially effective treatments.

**Table 1 (Abstract P04).** Myocarditis whose presentation mimics acute myocardial infarction: clinical and laboratory characteristics (*n* = 61)

Variables	Baseline	12 ± 3 months	*p* value
Age (years)	34.2 ± 7.1	–	
Male (%)	85.2	–	
Left ventricular ejection fraction	51.8 ± 5.2	55.3 ± 5.4	<0.01
Left ventricular ejection fraction <50 (%)	19.6	10.0	0.20
Left ventricular diastolic pressure abnormal (%)	14.7	8.3	0.39
EKG ST-T abnormal (%)	50.8	23.3	<0.01
Troponin (ng/ml; reference ≤0.05)	2.57 ± 1.47	–	
CK-MB (ng/ml; reference ≤3.6)	34.6 ± 23.8	–	
Segmental abnormality (%)	22.9	6.7	0.02

*CK-MB* creatine kinase MB, *EKG* electrocardiogram

### P05 Assessment of research productivity of published articles in the critical care unit of cardiac surgery patients

#### Arnaldo A da Silva^1,2^, Luis AA Costa^1^, Anderson CS Nascimento^1^, Paulo MS Souza^1^, Tatiane L Dias^1^, Mauricio R Pedrosa^1^, Walace S Pimentel Pimentel^1,2^, Regimar CM Ranzani^1^

##### ^1^UNIFESP – UTI Cirurgia Cardíaca/Hospital São Paulo, São Paulo, SP, Brazil; ^2^HIAE – Hospital Israelita Albert Einstein, São Paulo, SP, Brazil

**Background**

Cardiac surgery is a standard procedure worldwide. Many of the patients have been followed in the intensive care unit (ICU) after surgery. Many concerns about intensive care treatment could be solved by publication in the literature. Research is an essential and unique field that intensivists all over the world deal with, in addition to their daily clinical practice. Published articles represent a central part of the research process.

**Objective**

We aimed to assess the current quality of evidence from published papers in Scopus Database of the management of cardiac surgical patients in the critical care unit.

**Methods**

An internet electronic search was made in the Scopus database looking for published articles between 2004 and 2018. The search strategy involved core terms related to CCM and the specific search strategy was as follows: TITLE-ABS-KEY ( "cardiac surgery" ) OR TITLE-ABS-KEY ( "heart surgery" ) AND TITLE-ABS-KEY ( "intensive care" ) OR TITLE-ABS-KEY ( "critical care" ) AND TITLE-ABS-KEY ( adult ) AND NOT TITLE-ABS-KEY ( animal ) AND ( LIMIT-TO ( SRCTYPE , "j" ) ). There were no restrictions on language but the abstract should be written in English.

**Results**

A total of 4326 articles were identified using predefined search words. Almost all of them (4014) were published in English, with 1075 published in the United States. The five most important journals were *Journal of Cardiothoracic and Vascular Anesthesia* (impact factor (IF) = 0.2), *Annals of Thoracic Surgery* (IF = 1.2), *Critical Care Medicine* (IF = 3.88), *Journal of Thoracic and Cardiovascular Surgery* (IF = 2.26) and *European Journal of Cardio-Thoracic Surgery* (IF = 1.5). Overall, 112 systematic reviews have been published and only seven were published in the *Cochrane Database of Systematic Reviews* (IF = 6.22).

**Conclusions**

Many cardiac surgical patients have been monitored in the ICU, although a search on research on this has found low numbers of good papers and systematic reviews within this group of patients. Improving the quality of studies will provide high-quality intensive care management after cardiac surgery.

**References**

[1] Mador RL, Shaw NT. The impact of a Critical Care Information System (CCIS) on time spent charting and in direct patient care by staff in the ICU: a review of the literature. Int J Med Inform 2009;78(7):435–45.

[2] Zhang Z, Van Poucke S, Goyal H, Rowley DD, Zhong M, Liu N. The top 2,000 cited articles in critical care medicine: a bibliometric analysis. J Thorac Dis 2018;10(4):2437–47.

[3] Michalopoulos A, Bliziotis IA, Rizos M, Falagas ME. Worldwide research productivity in critical care medicine. Crit Care 2005;9(3):R258–65.

## Epidemiology

### P06 An evaluation of the influence of body mass index on severity scoring

#### Renato Chaves^1^, Rodrigo Deliberato^1^, Ary Neto^1^, Matthieu Komorowski^2^, David Stone^3^, Stephanie Ko^2^, Lucas Bulgarelli^1^, Carolina Ponzoni^1^, Leo Celi^2^, Alistair Johnson^2^

##### ^1^HIAE – Hospital Israelita Albert Einstein, São Paulo, SP, Brazil; ^2^Harvard-MIT Health Sciences & Technology, Cambridge, MA, USA; ^3^University of Virginia School of Medicine, Charlottesville, VA, USA

**Background**

Although one-third or more of critically ill patients in the United States are obese, obesity is not incorporated as a contributing factor in any of the commonly used severity of illness scores.

**Objective**

We hypothesize that selected severity of illness scores would perform differently if body mass index categorization was incorporated and that the performance of these score models would improve after consideration of body mass index as an additional model feature.

**Methods**

This retrospective study was exempt from institutional review board approval due to lack of direct patient intervention, and the security schema for which the re-identification risk was certified as meeting safe harbor standards by Privacert (Cambridge, MA; HIPAA Certification no. 1031219-2). We performed a secondary analysis of electronic health records from a multicenter ICU database, which contains de-identified data for more than 200,000 intensive care unit (ICU) admissions from 208 distinct ICUs across the United States between 2014 and 2015. All patient in the ICU database were eligible for inclusion, but only the first ICU admission of patients with documented height and weight were included. The primary outcome was in-hospital mortality.

**Results**

Of 108,402 patients from 189 different ICUs across the United States included in the analyses, 4661 (4%) were classified as underweight, 32,134 (30%) as normal weight, 32,278 (30%) as overweight, 30,259 (28%) as obese, and 9070 (8%) as morbidly obese. Baseline characteristics are summarized in Table 1. To assess the effect of adding body mass index as a risk adjustment element to the Acute Physiology and Chronic Health Evaluation (APACHE) IV and Oxford Acute Severity of Illness scoring (OASIS) systems, we examined the impact of this addition on both discrimination and calibration. We performed three assessments based upon: 1) the original scoring systems; 2) a recalibrated version of the systems; and 3) a recalibrated version incorporating body mass index as a covariate (Fig. 1). We also performed a subgroup analysis in groups defined using World Health Organization guidelines for obesity. Incorporating body mass index into the models provided a minor improvement in both discrimination and calibration. In a subgroup analysis, model discrimination was higher in groups with higher body mass index, but calibration worsened.

**Conclusions**

The performance of ICU prognostic models utilizing body mass index category as a scoring element was inconsistent across body mass index categories. Overall, adding body mass index as a risk adjustment variable led only to a minor improvement in scoring system performance.

**Table 1 (Abstract P06).** Demographic characteristics

Variable	Underweight (*n* = 4614)	Normal weight (*n* = 32,134)	Overweight (*n* = 32,278)	Obese (*n* = 30,259)	Morbidly obese (*n* = 9070)
Age (years), median (IQR)	68 (54–80)	67 (53–80)	66 (53–73)	64 (53–73)	60 (49–78)
Male, *n* (%)	1948 (41.8)	17,168 (53.4)	19,486 (60.4)	16,631 (55.0)	3776 (41.6)
BMI (kg/m^2^), median (IQR)	17.1 (16.0–17.9)	22.5 (20.9–23.8)	27.3 (26.1–28.6)	33.4 (31.5–35.9)	44.6 (41.8–49.3)
CCI, median (IQR)	4 (2–6)	4 (2–6)	3 (2–5)	3 (2–5)	3 (1–5)
ICU admission diagnosis, *n* (%)					
Sepsis (including pneumonia)	873 (18.7)	4936 (15.3)	4169 (12.9)	3967 (13.1)	1549 (17.1)
Cardiovascular disease	677 (14.5)	6336 (19.7)	8383 (25.9)	8303 (27.4)	2080 (22.9)
Other respiratory condition	750 (16.1)	2929 (9.1)	2344 (7.2)	2466 (8.1)	1159 (12.7)
Neurological condition	549 (11.7)	5008 (15.6)	5063 (15.7)	4551 (15.0)	1089 (12.0)
Renal condition	88 (2.0)	562 (2.0)	599 (2.0)	683 (2.0)	280 (3.0)
Medical reason for ICU admission, *n* (%)	4003 (85.9)	26,105 (81.2)	25,081 (77.7)	23,379 (77.3)	7412 (81.7)
SOFA score, median (IQR)	5 (2–7)	4 (2–7)	4 (2–7)	4 (2–7)	5 (2–7)
APACHE IV score, median (IQR)	56 (42–74)	53 (39–70)	50 (37–67)	49 (36–66)	49 (35–67)
OASIS score, median (IQR)	31 (25–38)	29 (23–37)	28 (22–35)	28 (22–35)	29 (23–36)
ICU LOS (days), median (IQR)	1.9 (1.0–3.3)	1.8 (0.9–3.1)	1.7 (0.9–3.1)	1.8 (1.0–3.3)	1.9 (1.0–3.7)
Hospital LOS (days), median (IQR)	5.8 (3.1–9.7)	5.2 (2.8–9.1)	5.1 (2.8–8.9)	5.2 (2.9–9.2)	5.7 (3.1–9.9)
Hospital mortality, *n* (%)	711 (15.3)	3390 (10.5)	2778 (8.6)	2437 (8.1)	772 (8.5)

Underweight = body mass index (BMI) <19.5 kg/m^2^, normal weight = BMI 18.5 to <25 kg/m^2^, overweight = BMI 25 to <30 kg/m^2^, obese = BMI 30 to <40 kg/m^2^, morbidly obese = BMI ≥40 kg/m^2^

*APACHE* Acute Physiology and Chronic Health Evaluation, *CCI* Charlson Comorbidity Index, *ICU* intensive care unit, *IQR* interquartile range, *LOS* length of stay, *OASIS* Oxford Acute Severity of Illness Score, *SOFA* Sequential Organ Failure Assessment

**Fig. 1 (Abstract P06).**
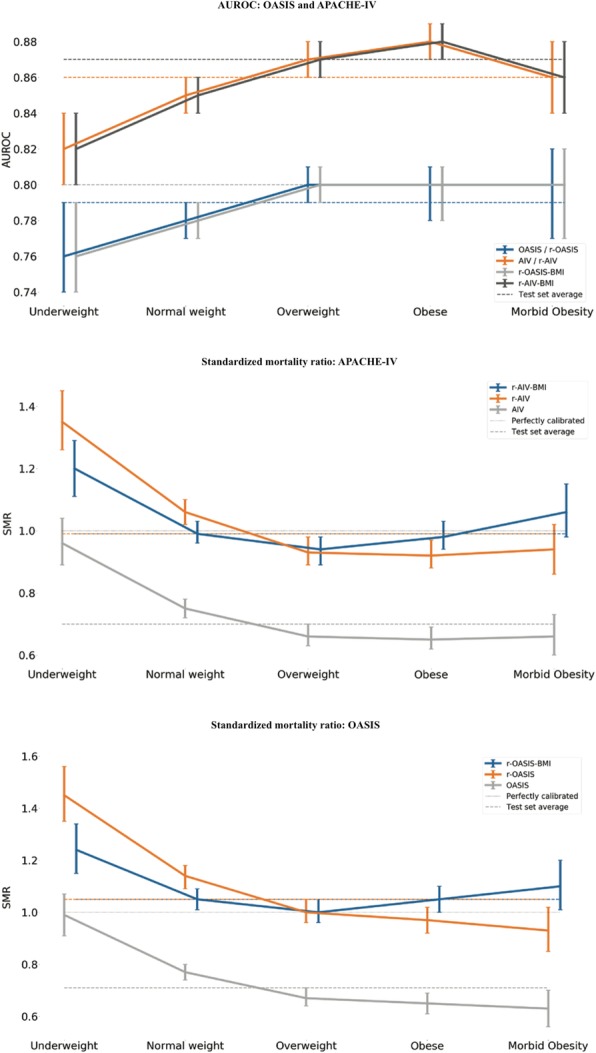
Area under the receiver operator characteristic (AUROC) and standardized mortality ratio (SMR) for Acute Physiology and Chronic Health Evaluation (APACHE) and Oxford Acute Severity of Illness Score (OASIS)

### P07 Clinical characteristics, risk factors, and outcome of chronic critical patients in a university hospital

#### Meriele Morete Capeletti, Giovanna Cristina Spagnuolo Brunello, Jair de Jesus Junior, Giovana Chiquetti, Marina Martines da Costa, Amanda Arantes Vieira, Fernanda Midori Kaneshima, Ulisses Enrique Colonheze, Josiane Festti, Claudia Maria Dantas de Maio Carrilho, Lucienne Tibery Queiroz Cardoso, Cintia Magalhâes Carvalho Grion

##### UEL – Universidade Estadual de Londrina, Londrina, Parana, Brazil

**Background**

The evolution of critical care has increased the number of patients who survive the acute insult but who remain with some organic dysfunction. Patients with this phenomenon are called chronic critically ill patients (CCIP). This condition is a serious and growing health problem in countries, resulting in high costs to the health systems.

**Objective**

We aimed to describe the incidence of CCIP, the clinical and epidemiological aspects of CCIP, and the risk factors for chronicity of critically ill intensive care patients at a university hospital.

**Methods**

This was a retrospective longitudinal study conducted from 2009 to 2016 in an intensive care unit (ICU) of the University Hospital Londrina. Patients were divided into two groups: chronic critical patients and acute critical patients. The variables analyzed as independent risk factors for critical patient chronicity were age, sex, single and multiple vasoactive drug use, hemodialysis, mechanical ventilation at admission, chronic disease, and type of admission category (medical, elective, and emergency post-operative). The variables evaluated as risk factors for hospital death among CCIP were age, sex, Acute Physiology and Chronic Health Evaluation (APACHE) II, Therapeutic Intervention Scoring System-28 (TISS 28), Sequential Organ Failure Assessment (SOFA), the presence of sepsis, mechanical ventilation for at least 96 h, tracheostomy, and being neurocritical.

**Results**

During the study period, 5044 patients were included of which 31.8% were chronic critical patients and 68.2% were acute critical patients. There was a trend towards an increase in chronic critical patient incidence over the study years. The risk factors for chronicity were male (odds ratio (OR) 1.18, 95% confidence interval (CI) 1.02–1.36), single vasoactive drug use (OR 1.37, 95% CI 1.17–1.61), mechanical ventilation at admission (OR 6.91, 95% CI 5.70–8.38), and medical type of admission (OR 1.45, 95% CI 1.24–1.70). Among chronic critical patient risk factors for hospital death were age (1.04, 95% CI 1.03–1.04), APACHE II score (1.05, 95% CI 1.04–1.07), mechanical ventilation for at least 96 h (3.89; 95% CI 2.16–7.00), and tracheostomy (1.35, 95% CI 1.06–1.71). The most frequently diagnosed conditions in the chronic critical patient were sepsis (47.4%), tracheostomy (45.0%), and mechanical ventilation for at least 96 h (95.6%).

**Conclusions**

We observed a high incidence of chronic critical patients in the present study and a trend of increasing incidence over the years. The conditions predominantly found in the chronic critical patient were prolonged mechanical ventilation, and the presence of tracheostomy and sepsis. The risk factors for chronicity were male gender, single vasoactive drug use, mechanical ventilation at admission, and medical type of admission. The risk factors for death of the chronic critical patient were age, APACHE II score, mechanical ventilation for at least 96 h, and the presence of tracheostomy.

### P08 Risk factors for ICU mortality among critically ill cancer patients at a public oncological ICU in São Luís, northeast Brazil

#### Ana Paula Pierre Moraes^1,2^, Gustavo Teixeira Alves^1^, José Ricardo Santos Lima^1^, Antônio Augusto Moura da Silva^2^

##### ^1^HTLF – Hospital de Câncer do Maranhão Tarquinio Lopes Filho, São Luis, MA, Brazil; ^2^UFMA – Universidade Federal do Maranhão, São Luis, MA, Brazil

**Background**

Recent data suggest that severity and the number of organ failures, management complexity, and performance status have higher impact on intensive care unit (ICU) and hospital mortality among cancer patients than the underlying neoplasm disease [1–3].

**Objective**

We aimed to evaluate the risk factors for ICU mortality among cancer patients.

**Methods**

This was a retrospective study conducted at an 11-bed ICU of a public cancer hospital in São Luis, Maranhão. All cancer patients >18 years old requiring ICU stay >24 h from January 2016 to December 2017 are included. We evaluated demographic and clinical variables, and ICU support at admission and during ICU stay. The risk factors for ICU mortality were investigated through multiple logistic regression analysis.

**Results**

Out of 712 patients, 473 (66%) had solid locoregional tumors, 156 (22%) had solid metastatical tumors, and 83(12%) were onco-hematology patients. There were 334 (47%) admissions due to medical reasons, and 343 (48%) and 35 (5%) for postoperative care after elective and emergency surgery, respectively. The overall ICU mortality was 34%. At multivariate analysis, the independent risk factors for ICU mortality were admission due to medical reasons (odds ratio (OR) 2.9, 95% confidence interval (CI) 1.51–5.69), onco-hematological disease (OR 2.04, 95% CI 1.06–3.91), high Simplified Acute Physiology Score III (SAPS 3; OR 1.04, 95% CI 1.02–1.07) and Sequential Organ Failure Assessment (SOFA) score (OR 1.16, 95% CI 1.06–1.26) at admission, the need for mechanical ventilation (OR 2.01, 95% CI 1.26–3.20) and vasoactive drugs (OR 2.37, 95% CI 1.35–4.15) during ICU stay, and nosocomial ICU infection (OR 4.97, 95% CI 2.64–9.36) (Table 1).

**Conclusions**

Characteristics prior to and upon ICU admission were identified as risk factors for ICU mortality. The severity of acute illness at ICU admission suggests that prompt recognition of organ dysfunction, the possibility of early ICU referral, and the expansion of prevention strategies to reduce hospital-acquired infection could be important strategies for reducing ICU mortality rates.

**References**

[1] Soares M, Caruso P, Silva E, et al. Characteristics and outcomes of patients with cancer requiring admission to intensive care units: a prospective multicenter study. Crit Care Med 2010;38(1):9-15

[2] Puxty K, McLoone P. Quasim T, Kisella J, Morrison D. Survival in solid cancer patients following intensive care unit admission. Intensive Care Med 2014;40(10):1409-1428.

[3] Rosa RG, Tonietto TF, Duso BA, Maccari JG, Oliveira RP, Rutzen W, et al. Mortality of adult critically ill subjects with cancer. Respir Care 2017;62(5):615-622.

**Table 1 (Abstract P08).** Results of multiple logistic regression analysis^a^ – final model of risk factors for ICU mortality in oncological patients

Select variable	Crude OR	Adjusted OR	95% CI	*p* value
Medical admission	11.53	2.93	1.51–5.69	0.002
Onco-hematological disease	8.25	2.04	1.06–3.91	0.03
SAPS 3	1.09	1.04	1.02–1.07	0.001
SOFA score on first day	1.46	1.16	1.06–1.26	0.001
Nosocomial ICU infection	3.68	4.97	2.64–9.36	<0.001
Mechanical ventilation	2.31	2.01	1.26–3.20	0.003
Vasoactive drugs	5.84	2.37	1.35–4.15	0.003

*CI* confidence interval, *ICU* intensive care unit, *OR* odds ratio, *SAPS 3* Simplified Acute Physiology Score III, *SOFA* Sequential Organ Failure Assessment

^a^ Hosmer-Lemeshow χ^2^ *p* value = 0.80

### P09 The Brazilian public health system: the reality of heart failure

#### Elaine Fonseca^1^, Ana Cláudia Costa^1^, Paula Tolentino^1^, Gabriela Amaral^1^, Débora Braga^1^, Pedro Esaki^1^, Ana Couto^1^, Nathália Lima^1^, Giuliano Generoso^2^

##### ^1^UNICEPLAC – Centro Universitário do Planalto Central Apparecido Santos, Gama, DF, Brazil; ^2^HSL – Cardiology Center, Hospital Sírio Libanes, São Paulo, SP, Brazil

**Background**

Heart failure (HF) is characterized by the inability of the heart to pump effectively as a result of structural or functional cardiac modifications. HF is considered severe and the 5-year survival rate is about 35%. It is estimated that it affects 23 million people in the world, and in Latin America is significant due to its economic, social, and cultural aspects.

**Objective**

We aimed to evaluate the epidemiological profile of patients hospitalized in the Brazilian public health system (SUS) with heart failure in the last decade, as well as the mortality rate and costs to the government.

**Methods**

Our study is a cross-sectional, retrospective, observational study of data collected through the hospital information system of the public health system (SIH/DataSUS) from January 2008 to December 2017. The data from 2018 were excluded from the sample due to the possibility of updates.

**Results**

The annual average number of hospitalized patients with heart failure was 241,500, representing 21.28 ± 2.11% of hospital admissions for diseases of the circulatory system. Comparing the years 2008 and 2017, a gradual decline in hospitalization was seen, with 27.3% less in 2017. The standard cost of hospitalization increased progressively, reaching R$1,582,00 in 2017, 67% more than 2008; moreover, there was an additional 1 day spent in the hospital. When we categorized by the age group, people who were 80 years or older had a higher prevalence, but patients from 60 to 79 years old represent 50% of the sample. Regarding the difference between genders, men between 30 and 69 years old were hospitalized about 5% more; however, women correspond to 56.5% of those patients aged 80 years or more. There were annual increases in mortality, reaching 10.7% in 2017. Excluding patients younger than 5 years old, mortality increases as the age group rises, with those aged 80 years or more representing 14.41%, followed by those aged 70 to 79 years at 9.96%, with no significant difference between genders. Concerning the age group in which mortality is high (60 to 80 years or older) there was an increase in the number of hospitalizations with morbidities in the same period analyzed, such as acute myocardial infarction (79.49%) atherosclerosis (94.03%), and sepsis (297.07%). In the context of region, the southeast represents 42%, followed by the northeast and south, which together correspond to 45.5%.

**Conclusions**

Despite a significant decrease in hospital admissions as a consequence of HF, mortality increases every year, as well as the financial cost. The most affected patient profile has a higher prevalence of morbidities, suggesting a more severe clinical scenario.

### P10 Does every neurosurgical patient need intensive care unit treatment in the immediate postoperative period?

#### Antonio Pergentino Barreira Neto, Alana de Alcântara Brito, Raquel Lima Sampaio, Betina Santos Tomaz, Wênya Palácio Xavier de Melo, Francisco Albano de Meneses

HGF – Hospital Geral de Fortaleza, Fortaleza, CE, Brazil

**Background**

Although most neurosurgical procedures are considered to be high risk and with potential complications, immediate admission to the intensive care unit (ICU) is questioned.

**Objective**

In this study, we analyzed the patients admitted to a neurosurgical ICU, correlating pre-admission markers with the respective outcomes.

**Methods**

We analyzed data from 47 neurosurgical patients hospitalized at the Intensive Care Center of the General Hospital of Fortaleza from January to March 2019. In addition to the demographic data, we inferred severity through the Systemic Inflammatory Response Syndrome (SIRS) and the duration of surgical proceedings. The SPSS software program was used to process the statistical analysis.

**Results**

Of the total patients, 24 were men (51%), and the mean age was 50 ± 15 years, the length of stay was an average of 7 ± 10 days, and mortality was 5%. Surgeries were mainly (95%) elective. The main diagnoses were brain tumor (70.2%) and subarachnoid hemorrhage (19.1%). SIRS values ranged from 0 to 3 points, with <2 points in 72.3% of patients; 2 of 3 patients who died had only 1 point. In 70.2% of the patients the surgical procedure lasted around 3 and 6 hours. Using the Pearson chi-square test, the correlations had the following meanings: SIRS x duration of surgery, p: 0.153; SIRS x outcome, p: 0.470, and procedure duration x outcome, p: 0.829.

**Conclusions**

Given the low initial severity, inferred by the SIRS score, the low mortality and the absence of a significant correlation of surgical duration with the outcome, it seems rational to disregard an imperative admission to the ICU in the immediate postoperative window. In view of the high demand for ICU beds, costs, and ethical aspects, we suggest the creation of a unit specializing in post-neurosurgery.

### P11 Early and late mortality following discharge from the intensive care unit: a multicenter prospective cohort study

#### Regis Rosa, Maicon Falavigna, Evelin Sanchez, Daniel Sganzerla, Camila Dietrich, Denise de Souza, Gabriela Rech, Rosa dos Santos, Cassiano Teixeira

##### HMV –Hospital Moinhos de Vento, Porto Alegre, RS, Brazil

**Background**

Long-term mortality is higher in intensive care unit (ICU) survivors than in the general population, yet few data exist on the incidence, causes, and predictors of early and late mortality after ICU discharge.

**Objective**

We aimed to identify the incidence, causes, and predictors of early and late mortality among adult patients discharged alive from ICUs.

**Methods**

This was a multicenter prospective cohort study was performed in 10 tertiary hospitals in Brazil from May 2014 to December 2018. Adult ICU survivors with an ICU stay >72 h for medical and emergency surgical admissions or >120 h for elective surgical admissions were followed up for 12 months. The main outcomes were early (30 days) and late (31 to 365 days) mortality. Causes of death were independently extracted from death certificates and medical records by two critical care physicians and grouped according to the Tenth International Statistical Classification of Diseases and Related Health Problems.

**Results**

A total of 1554 patients were enrolled. Twelve-month cumulative mortality was 28.2% (439 deaths). The incidence of early mortality was 7.9% (123 deaths), and the incidence of late mortality was 22.3% (316 deaths). Most early and late deaths were caused by infections, followed by circulatory system diseases and neoplasms (Fig. 1). Multivariate analysis identified age ≥65 years (*p* = 0.016), pre-ICU high comorbidity (Charlson index, *p* = 0.008), pre-ICU physical dependence (Barthel index, *p*<0.001), risk of death at ICU admission (P=0.02), and any ICU-acquired infection (P<0.001) as predictors of early mortality. Age ≥65 years (P=0.028), pre-ICU high comorbidity (P<0.001), pre-ICU physical dependence (P<0.001), and risk of death at ICU admission (P<0.001) were predictors of late mortality.

**Conclusions**

In this multicenter prospective cohort study performed with adult ICU survivors, infection was the main cause of early and late deaths. Older age, pre-ICU comorbidities and physical dependence, and severity of critical illness were associated with an increased risk of early and late mortality after ICU discharge, while ICU-acquired infections were associated with an increased risk of early mortality. These findings may guide future interventions aimed to prevent post-ICU deaths.

**Fig. 1 (Abstract P11).**
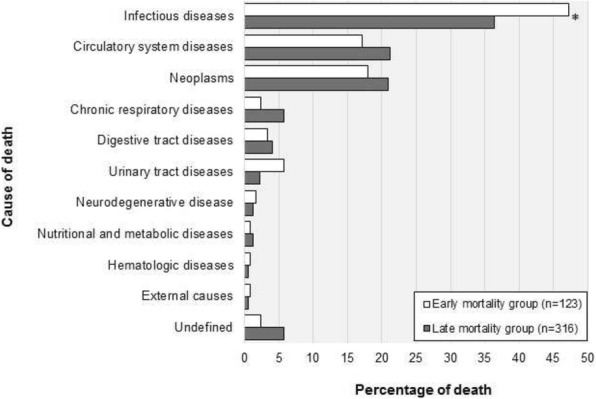
Causes of early and late deaths after ICU discharge. *p = 0.04

### P12 SAPS 3 performance in very elderly patients admitted to an intensive care unit in Brazil

#### Leandro Utino Taniguchi^1,2^, Ellen MP Siqueira^1^

##### ^1^HSL – Hospital Sírio Libanês, São Paulo, SP, Brazil; ^2^HCFMUSP – Hospital das Clinicas da USP, São Paulo, SP, Brazil

**Background**

The Simplified Acute Physiology Score III (SAPS 3) is the most accurate prognostic model for Brazil [1]. However, its performance in very elderly Brazilian patients (age ≥80 years) has not been adequately evaluated.

**Objective**

We aimed to compare the performance of SAPS 3 in patients older than 80 years with those younger than 80 years.

**Methods**

This was a retrospective cohort analysis of the Epimed® administrative database of adult patients (>18 years) on first admission to the intensive care unit (ICU) of Hospital Sírio Libanês (São Paulo, Brazil) from 2012 to 2016. Patients were categorized according to age (≥80 years or younger). We evaluated discrimination using the area under the receiver operating characteristic curve (AUROC) and the agreement between observed and expected mortality (calibration) using the calibration belt.

**Results**

We studied 7468 patients (age 65.9 ± 18.3 years, 53.3% male, SAPS 3 score 40.4 ± 13.4, 41.4% had cancer, 11.2% died during hospitalization), with 2002 patients (26.8%) older than 80 years. SAPS 3 discrimination was better for elderly patients below 80 years of age (AUROC = 0.81) than for very elderly patients over 80 years of age (AUROC = 0.70) (*p* < 0.001; Fig. 1). After we applied the calibration belt we observed that, in those aged 80 years and above, SAPS 3 underestimated mortality in those with low risk and overestimated it in those with middle risk . In those less than 80 years of age, SAPS 3 underestimated mortality only in those with low risk (Fig. 2).

**Conclusions**

SAPS 3 has some limitations in those patients older than 80 years of age.

**Reference**

[1] Moralez GM, Rabello LSCF, Lisboa TC, Lima MDFA, Hatum RM, De Marco FVC, et al. External validation of SAPS 3 and MPM0-III scores in 48,816 patients from 72 Brazilian ICUs. Ann Intensive Care 2017;7:53.

**Fig. 1 (Abstract P12).**
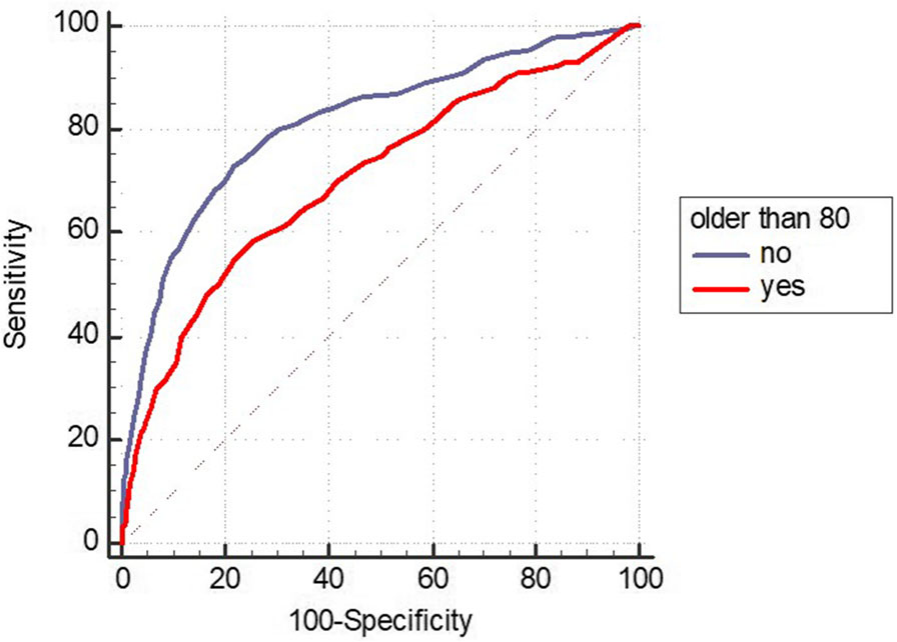
Receiver operating characteristic curve of the Simplified Acute Physiology Score III (SAPS 3) discrimination between those patients older than 80 years and those below 80 years of age

**Fig. 2 (Abstract P12).**
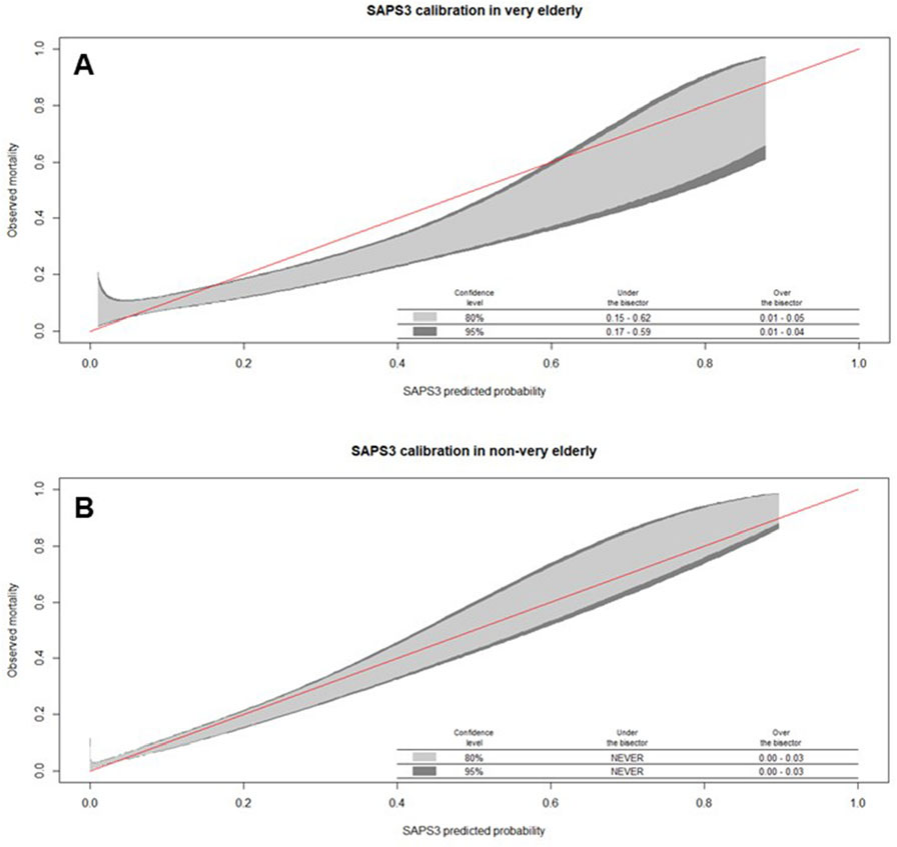
Calibration belt for the Simplified Acute Physiology Score III (SAPS 3) in those patients (A) older than 80 years or (B) not older than 80 years

## Hemodynamics/Shock

### P13 Conduct in emergency medicine for new anticoagulants at surgeries and hemorrhagic events

#### Carolina Lopes, Marcela Hungaro, Hélio Guimarães

CUSC – Centro Universitário São Camilo, São Paulo, SP, Brazil

**Background**

The need to control thrombotic disorders has led to the development of new oral anticoagulants (NOACs), such as dabigatran, rivaroxaban, and apixaban. According to each pharmacokinetic, emergency physicians must be able to use strategies that revert the anticoagulant effect to deal with the urgent care.

**Objective**

The aim of this study was to undertake a systematic review of the literature to display the conduct indications for the population in the use of NOACs during hemorrhagic events or urgent surgeries.

**Methods**

This article is a systematic review carried out from the descriptors "new anticoagulants" AND "emergency" on the Bireme data network where, from 335 scientific articles, 12 followed the inclusion criteria. Were selected the full research in LILACS, MEDLINE, and IBECS databases, published between 2010 and 2018, in Portuguese or English and that had as the main subject “Anticoagulants” and “Emergency Hospital Service”, excluding papers characterized as case reports.

**Results**

The surgical recommendation for the use of NOACs for a patient is when the plasma concentration of the drug is below 30 ng/ml. If the stipulated amount is exceeded, the indication is to postpone the surgery and to dose again after 12 h. In patients with compromised renal function, hemodialysis should be considered to reduce the level of the drug. When a postponement is not possible, it is prescribed to follow the procedure and, in case of abnormal bleeding, to administer an unspecific hemostatic factor (PCC or FEIBA), or the only specific hemostatic factor on the market, idarucizumab, for those who use dabigatran. In minor bleeding events, general actions for control and the delay of the anticoagulant are recommended. In major events, the following are considered: localization of the bleeding source; hydric control and maintenance of renal function; transfusion; use of activated charcoal; dialysis; and use of hemostatic agents. The immediate neutralization of the anticoagulant effect in intracranial hemorrhage is emphasized. In extended bleedings with plasma concentrations below 30 ng/ml, it is considered improbable that the drug provoked the occurrence, and reversion should not be carried out except when other actions cannot be performed. Finally, due to a lack of research, these recommendations cannot be taken as absolute guidelines but they can be a strategy against scarce scientific evidence.

**Conclusions**

A large part of the literature within this area is based on the opinions of professionals, which focuses on evidence level 5. Thus, the designation of specific measures has become necessary, and an evolution of the guidelines is expected.

### P14 Intensive approach to systemic lupus erythematosus associated with preeclampsia and its implications in a concept: a case report

#### Bruno Vinicius Castello Branco^1^, Hugo Correia De Andrade Urbano^2^, Laura Carolina Menezes Vieira Silva^1^, Lucas Carvalho^2^, Luiz Alberto Wanderley De Menezes Silva^1^, Matheus Victor Pereira^1^, Tayná Alves dos Santos^1^

##### ^1^UFMG – Universidade Federal de Minas Gerais, Belo Horizonte. MG, Brazil; ^2^HVS – Hospital Vila da Serra, Belo Horizonte, MG, Brazil

**Background**

Preeclampsia is one of the most frequent complications of pregnancy in individuals with systemic lupus erythematosus (SLE), occurring in 16–30% in this group compared with 4.6% in the general obstetric population. HELLP syndrome is a possible outcome, presenting a challenging prognosis.

**Objective**

We aimed to report a case of uncontrolled SLE and its repercussion on pregnancy, which includes eclampsia, HELLP syndrome, fetal loss, and ischemic stroke.

**Case presentation**

A female patient, 28 years old, white, pregnant for the first time (22 weeks) and an SLE carrier attended our service reporting headache, abdominal pain, high blood pressure (HBP), and emesis; following the use of metoclopramide, the patient presented with lowering of sensory function. Although we used sulfate, she had a seizure (eclampsia). Evidence of ischemic stroke (Fig. 1) was confirmed by a brain magnetic resonance imaging (MRI). The laboratory review showed a diagnosis of HELLP (Table 1). Pulse corticosteroid therapy with methylprednisolone, plasmapheresis with albumin, and doses of acetylsalicylic acid, simvastatin, and phenytoin were initiated. In addition, the induction of delivery began. At the time of writing, on the 29th day of hospitalization, the patient was conscious, despite presenting neurological deficits.

**Discussion**

High estrogen levels during pregnancy lead to an increase in body cytokines enhancing the action of SLE antibodies against cell membranes, which stimulates thrombogenesis and intensifies preeclampsia by damaging the endothelium and the platelets. Such damage causes HBP through vasoconstriction. Intensive treatment requires pregnancy interruption, immune system control, and neurological stabilization.

**Conclusions**

SLE implies potential risks for the pregnant woman and the fetus. Therefore, it is important to monitor these patients to investigate any clinical or laboratory changes and, thus, intercede for a better prognosis.

**Consent to publish**

Informed consent to publish has been obtained from the patient’s family.

**Table 1 (Abstract P14).** Patient results and cut-off points for HELLP syndrome

	LDH (IU/l)	TB (mg/dl)	AST (U/l)	ALT (U/l)	Platelets (U/μl)
Cut-off point	>600	>1.2	>70	>50	<100,000
Day 1	6660	3.49	1169	439	75,000
Day 5	1067	0.59	357	724	37,000
Day 10	411	0.67	31	35	17,000
Day 29	–	–	–	–	180,000

(1) Lactate dehydrogenase (LDH) and/or total bilirubin (TB) upper levels, (2) aspartate aminotransferase (AST) and/or alanine aminotransferase (ALT) upper levels, and (3) platelet lower levels

**Fig. 1 (Abstract P14).**
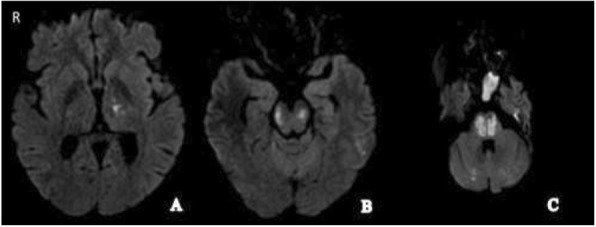
Magnetic resonance diffusion-weighted images showing high signal foci in (A) the left basal ganglia, (B) in the midbrain, and (C) in the pons and the cerebellum

## Infection

### P15 Is there any difference between patients receiving guided and empirical therapy with polymyxins?

#### Thalita Talizin^1,2^, Camila Lopes^2^, Isabella Azevedo^2^, Késia Paes^2^, Leticia Silva^2^, Marcos Tanita^2^, Karine Boll^2^, Claudia Carrilho^2^, Lucienne Cardoso^2^, Cintia Grion^2^, Eduardo Medeiros^1^

##### ^1^UNIFESP – Universidade Federal de São Paulo, São Paulo, SP, Brazil; ^2^UEL – Universidade Estadual de Londrina, Londrina, PR, Brazil

**Background**

Polymyxins are last-line therapy for treatment of multidrug-resistant bacteria. There is a lack of knowledge of how to use polymyxins, especially in the critically ill who often are given it empirically and have high rates of death [1].

**Objective**

We aimed to compare patients who received guided and empirical therapy with polymyxins during intensive care unit (ICU) stay.

**Methods**

We looked at a historical cohort of all consecutive patients who were given polymyxins to treat ventilator-associated pneumonia from 1 January 2017 to 31 January 2018 during hospitalization in an ICU from a university hospital endemic for carbapenem-resistant bacteria. This study was approved by Universidade Federal de São Paulo’s Ethics Board, approval number 95308418.5.0000.5505. Statistical analyses were performed using MedCalc for Windows, version 18.9 (MedCalc Software, Ostend, Belgium) and the significance level adopted was 0.05.

**Results**

There were 179 patients with a median time of treatment of 11 days (interquartile range (IQR) 5.0–14.75). There was no difference between empirical and guided therapy regarding mortality or prognostics scores (Table 1). Microbiological confirmation occurred in 47 cases (73.4%) from patients who had initial empirical treatment. *Acinetobacter baumannii* was the etiological agent in 121 (67.6%) cases. Combination therapy occurred in 165 cases (92.2%), mostly with carbapenem (51.5%). In-hospital mortality was 81%. Multivariate analysis showed that Sequential Organ Failure Assessment (SOFA) score on the day polymyxin was prescribed (odds ratio (OR) 1.2, 95% confidence interval (CI) 1.02–1.42; *p* = 0.03) and comorbidities (OR 7.5, 95% CI 3.12–17.81; *p*<0.0001) as risk factors for mortality.

**Conclusions**

There was no statistical difference between patients using guided and empirical treatment regarding mortality and prognostic scores.

**Reference**

[1] Tsuji BT, Pogue JM, Zavascki AP, et al. International consensus guidelines for the optimal use of the polymyxins: endorsed by the ACCP, ESCMID, IDSA, ISAP, SCCM, and SIDP. Pharmacotherapy 2019;39(1):10–39.

**Table 1 (Abstract P15).** Distribution of clinical data stratified between guided and empirical therapy

	All patients (*n* = 179)	Guided therapy (*n* = 115)	Empirical therapy (*n* = 64)	*p* value
Mortality, *n* (%)	145 (81.0)	90 (78.3)	55 (85.9)	0.21*
SAPS 3, mean (SD)	68.5 (16.7)	67.7 (17.0)	70.0 (16.2)	0.38^†^
SOFA, median (IQR)	7 (6–10)	7 (6–10)	8 (6–11)	0.27^‡^

*IQR* interquartile range, *SAPS 3* Simplified Acute Physiology Score 3, *SD* standard deviation, *SOFA* Sequential Organ Failure Assessment

* Chi-square test

^†^ Student’s *t* test

^‡^ Mann–Whitney test

### P16 Risk factors for mortality among patients with carbapenem-resistant *Klebsiella pneumoniae* infection: a retrospective study

#### Fernanda Nunes Passos^1^, Clara Sá Macedo Dantas^1^, Juliana Caldas Ribeiro Bittencourt^1,2^, Rogerio da Hora Passos^1,2^, João Gabriel Rosa Ramos^2^, Ana Verena Almeida Mendes^1,2^, Maria Goreth Matos de Andrade Barberino^2^, Márcio de Oliveira Silva^2^, Camila Araujo de Lorenzo Barcia^2^, Andre Luiz Nunes Gobatto^2^, Lis Kalid^2^, Suzete Nascimento Farias da Guarda^2,3^, Paulo Benigno Pena Batista^2^

##### ^1^EBMSP – Escola Bahiana de Medicina e Saúde Pública, Salvador, Bahia, Brazil; ^2^HSR – Hospital São Rafael, Salvador, Bahia, Brazil; ^3^UFBA – Universidade Federal da Bahia, Salvador, Bahia, Brazil

**Background**

Carbapenem-resistant *Klebsiella pneumoniae* (CR-KP) infection has become a worldwide problem.

**Objective**

The aim of this study was to identify risk factors for 28-day mortality in patients with CR-KP infection.

**Methods**

We conducted a retrospective study of CR-KP infections in critically ill patients between January 2015 and October 2017 at the Hospital São Rafael. Continuous variables were compared by the Student *t* test and categorical variables by the χ^2^ test. Multivariate logistic regression analysis was performed to determine the independent risk factors for mortality. The study was approved by Hospital São Rafael‘s Ethics Board, approval number 2890446.

**Results**

A total of 90 patients with CR-KP infections were identified. Twenty-eight-day mortality was 40.0% (*n* = 36). Variables associated with 28-day mortality in the univariate analysis are shown in Table 1. Charlson Comorbidity Index (CCI; *p* = 0.001; odds ratio (OR) 1.38, 95% confidence interval (CI) 1.14–1.68) and Sequential Organ Failure Assessment (SOFA; *p* = 0.014; OR 1.33, 95% CI 1.06–1.66) were independently associated with mortality in the multivariate analysis.

**Conclusions**

In critically ill patients infected with CR-KP, 28-day mortality was independently associated with SOFA and CCI.

**Table 1 (Abstract P16).** Characteristics of patients with infections caused by carbapenem-resistant *Klebsiella pneumoniae*

Variable	Survived (*n* = 54)	Died (*n* = 36)	*p* value
CCI	3.6 ± 3.1	6.4 ± 3.3	0.000
ICU admission	33 (61.1)	33 (91.7)	0.001
Sepsis	25 (46.3)	25 (69.4)	0.030
SOFA score	2.2 ± 2.4	4.7 ± 3.4	0.000
Infection source			0.162
Pneumonia	1 (1.9)	2 (5.6)	
Intra-abdominal	7 (13.0)	4 (11.1)	
Urinary tract	26 (48.1)	8 (22.2)	
Surgical wound	3 (5.6)	1 (2.8)	
Bacteremia	14 (25.9)	14 (38.9)	
Catheter-related	1 (1.9)	2 (5.6)	
Soft tissue	2 (3.7)	4 (11.1)	
Other	0	1 (2.8)	
Empirical antibiotic therapy			0.020
Inadequate	47 (87.0)	21 (58.3)	
Monotherapy	1 (1.9)	3 (8.3)	
Two-drug combination	3 (5.6)	7 (19.4)	
Three-drug combination	3 (5.6)	5 (13.9)	

Data are expressed as *n* (%) or mean ± standard deviation

*CCI* Charlson Comorbidity Index, *ICU* intensive care unit, *SOFA* Sequential Organ Failure Assessment

### P17 Urinary tract infections antibiotic resistance in a community in Juiz de Fora, Brazil

#### Luiz AA Costa^1,2^, Rosangela MC Cunha^2^, Carlos OFF Candido^2^, Marina A Delmonte^2^, Arnaldo A Silva^1,3^, Regimar CM Ranzani^1^, Walace S Pimentel^1,3^

##### ^1^HSP/UNIFESP – UTI Cirurgia Cardíaca/Hospital São Paulo, São Paulo, SP, Brazil; ^2^FCMS-JF/SUPREMA – Faculdade de Ciências Médicas e da Saúde de Juiz de Fora, Juiz de Fora, Brazil; ^3^HIAE – Hospital Israelita Albert Einstein, São Paulo, SP, Brazil

**Background**

Antibiotic resistance is now common in urinary tract infections (UTIs). The number of UTIs caused by drug-resistant bacteria is increasing, and new data show an approximate 25–30% resistance to oral drugs used to treat UTIs. Because of the way bacteria are spread, resistance patterns vary by region and are more severe in some places than in others.

**Objective**

This study will analyze the antimicrobial resistance pattern of agents causing UTI in the general population of Juiz de Fora, Brazil.

**Methods**

This is an ecological study (descriptive, cross-sectional, and observational) in which urine culture and antibiogram records were analyzed from two large laboratories in the city of Juiz de Fora, Minas Gerais, Brazil, containing community group patients from August 2016 to January 2017. We have excluded: urine samples from patients outside the period mentioned above; samples of nonresidents in Juiz de Fora; samples of hospitalized patients; demographic samples illegible or characterized by imprecise information; and samples with an inconclusive antibiogram.

**Results**

Of 3118 samples analyzed, 603 (19.3%) presented a positive urine culture result and matched the inclusion criteria of the present study. In this group, *Escherichia coli* appeared as the most prevalent organism, and was present in 364 of the positive tests (60.4%). Furthermore, 179 (49%) were resistant to ampicillin, 126 (36%) were resistant to sulfamethoxazole-trimethoprim, 83 (23%) were resistant to ciprofloxacin, and 84 (23.5%) were resistant to norfloxacin. Therefore, the demographic groups of the global sample are positive, and no statistically different resistance patterns were observed between groups in the study sample.

**Conclusions**

*E. coli* and *K. pneumoniae* are the most common germs seen in the study. Broad-spectrum antibiotics are the only alternative with a better sensibility for UTIs in this community. As UTI-causing bacteria become more resistant to available antibiotics, the need to explore new strategies for managing UTIs is clear. Further studies are required to establish a global antimicrobial resistance program and avoid treatment failure.

**References**

[1] Ohieku JD, Magaji RA. Urinary tract infections associated with Escherichia coli: a 2005 to 2009 clinical assessment of trends in fluoroquinolones activities in Maiduguri-City, Nigeria. JAPS 2013;3(8):84–91.

[2] Cunha MA, Assunção GLM, Medeiros IM, Freitas MR. Antibiotic resistance patterns of urinary tract infections in a northeastern Brazilian capital. Rev Inst Med Trop Sao Paulo 2016;58:2.

[3] Fasugba O, Gardner A, Mitchell BG, Mnatzaganian G. Ciprofloxacin resistance in community- and hospital-acquired Escherichia coli urinary tract infections: a systematic review and meta-analysis of observational studies. Infectious Diseases 2015;15:545.

[4] Dickstein Y, Geffen Y, Andreassen S, Leibovici L, Paul M. Predicting antibiotic resistance in urinary tract infection patients with prior urine cultures. Antimicrob Agents Chemother 2016;60(8):4717–21.

### P18 Evaluation of the sensitivity and specificity of the molecular method of diagnosis of *Mycobacterium tuberculosis* for smear microscopy and culture-based methods

#### Leandro Martins Lima Ronaldo Rodrigues Costa

##### UFJF – Universidade Federal de Juiz de Fora, Juiz de Fora, Brazil

**Background**

The Xpert MTB/RIF® assay (Cepheid, USA) is currently the World Health Organization (WHO)-recommended rapid diagnostic technique for tuberculosis most commonly used by countries around the world. The molecular methodology used in this test is a semi-nested real-time polymerase-chain-reaction (PCR), with the purpose of amplifying a specific sequence of the *Mycobacterium tuberculosis* (MTB) *rpoB* gene and identifying mutations in the rifampicin (RIF) resistance-determining region that detects both MTB and RIF resistance.

**Objective**

The objective of this study was to evaluate the sensitivity and specificity of the molecular method recommended by the WHO in relation to classical laboratory diagnostic methods for tuberculosis, based on the comparative culture of mycobacteria.

**Methods**

For this study, 664 clinical samples were obtained from the clinical laboratory of the João Penido Hospital, Juiz de Fora, MG, Brazil, processed during routine laboratory work between January 2015 and June 2018. Among the samples, 638 were of pulmonary origin and 26 were of extrapulmonary origin. All samples included in the study were processed by three methodologies: smear microscopy by the Ziehl-Neelsen method (ARB) , sowing in culture medium (BKC), and nested real-time PCR (RMT). Considering the diagnosis by BKC as the gold standard for disease detection, this technique was taken as the basis for comparison between molecular and bacilloscopic methods, as well as in the methodologies employed by several authors. Considering the obtained results, parameters of sensitivity, specificity, positive predictive value (PPV), and negative predictive value (NPV) were calculated for ARB and RMT.

**Results**

Bacilloscopy identified 148 positive tests, which gave it a sensitivity of 83.6%; however, in a total of 169 bacilloscopies performed with positive result, 21 positive results were related to negative cultures, generating a PPV of 87.6%, higher than the PPV calculated for the RMT. The specificity was calculated in 95.3%. With this, the NPV was calculated in 93.6%, since 29 positive cultures were not detectable by smear. For pulmonary samples, 169 detectable results of RMT were observed in 174 BKC positive for MTB, revealing a test sensitivity of 97.1%. The PPV was calculated in 75.5%, since 55 samples with negative BKC showed detectable RMT. The specificity of RMT was 87.4%, considering that 382 samples presented negative results in 437 negative cultures, but the NPV was 98.7%, since in 387 undetectable results only five presented a positive culture.

**Conclusions**

Considering the results presented, the superiority of RMT in relation to bacilloscopy is clear considering the sensitivity of the test; however, due to this high sensitivity, it is recommended to use RMT for the primary diagnosis to avoid false positive results as could be observed in low PPV in relation to smear microscopy. Finally, RMT is a complementary technique to the diagnosis of tuberculosis and is not a substitute technique for those already used.

## Nephrology

### P19 Risk factors of intradialytic hypotension in critically ill patients undergoing intermittent dialysis

#### Rogério Passos^1,2^, Érica Melo^1^, Aline Pontara^1^, João Gabriel Ramos^1^, Juliana Caldas^1^, Octavio Messeder^2^, Augusto Farias^2^, Maria Fernanda Coelho^2^

##### ^1^HSR – Hospital São Rafael, Salvador, Bahia, Brazil; ^2^HP – Hospital Português da Bahia, Salvador, Bahia, Brazil

**Background**

Intradialytic hypotension (IDH) is one of the most common reasons for modification of the dialysis prescription from the intermittent to the continuous method. It is a barrier for effective treatment in critically ill patients and can also contribute to a delay in recovery of renal function and improvement in other organ failures. However, fluid status remains poorly defined by clinical examination findings, and sonography is a noninvasive method that has been proposed to enhance the bedside assessment of intravascular fluid volume.

**Objective**

The aim of this study was to determine the prevalence and risk factors of IDH in critically ill patients undergoing intermittent dialysis. We also sought to evaluate different profiles based ion sonographic findings just before the initiation of hemodialysis and their relation to IDH.

**Methods**

From January 2016 to April 2018, a prospective study was conducted in a single center with 248 patients with acute renal injury with an indication for intermittent dialysis.

**Results**

Hypotension was seen in 31.9% of patients and 28-day overall mortality occurred in 50 patients. Among the factors significantly associated with hypotension were high levels of lactate and low mean arterial pressure. Of the patients, 65.8% had a diagnosis of sepsis, 40.5% were using noradrenaline, and 25.3% required mechanical ventilation.

**Conclusions**

We identified risk factors related to fluid status and vasomotor tone that may be implicated in the occurrence of IDH. However, further studies should be considered to validate these results and design an appropriate specific treatment.

**Table 1 (Abstract P19).** Distribution of parameters according to the occurrence of hypotension univariate analysis

	Hypotension		
Variable	No	Yes	*p* value
	*n* = 169 (68.1%)	*n* = 79 (31.9%)	
Gender			0.684
Male, *n* (%)	103 (60.9)	46 (58.2)	
Female, *n* (%)	66 (39.1)	33 (41.8)	
Age (years)	66 (55–76)	70 (64–76)	0.018
Charlson score	10 (8–12)	10 (8–12)	0.116
APACHE score	14 (12–18)	16 (13–18)	0.215
SOFA score	8 (6–10)	8 (7–10)	0.057
Sepsis, *n* (%)	71 (42)	52 (65.8)	<0.001
Use of norepinephrine, *n* (%)	5 (3)	32 (40.5)	<0.001
Mechanical ventilation, *n* (%)	14 (8.3)	20 (25.3)	<0.001
Hydric balance (ml)	1800 (1535–2320)	1750 (1540–2350)	0.762
Ultrasound data, *n* (%)			
B line	106 (62.7)	38 (48.1)	0.030
Cava vein collapse	14 (8.3)	60 (75.9)	<0.001
Profile, *n* (%)			<0.001
A	100 (59.2)	8 (10.1)	
B	8 (4.7)	30 (38)	
C	6 (3.6)	30 (38)	
D	55 (32.5)	11 (13.9)	
Lactate (mmol/l)	1.3 (1.1–1.7)	1.7 (1.2–2.2)	<0.001

Values are shown as median (range) unless otherwise indicated

*APACHE* Acute Physiology and Chronic Health Evaluation, *SOFA* Sequential Organ Failure Assessment

**Table 2 (Abstract P19).** Multivariate logistic regression analysis of the univariate variables of the patients with hypotension

Variable	Parameter estimated	Standard error	Odds ratio	95% CI	*p* value
Age	0.018	0.015	1.018	0.989–1.049	0.228
Blood flow rate	0.006	0.007	1.006	0.993–1.019	0.379
Dialysate flow rate	–0.007	0.004	0.993	0.984–1.001	0.096
Ultrafiltration	0.001	0.004	1.000	1.000–1.001	0.136
Mean blood pressure	–0.039	0.015	0.962	0.934–0.991	0.010
Lactate	–0.187	0.398	0.829	0.380–1.810	0.638
Use of norepinephrine	2.736	0.679	15.425	4.078–58.353	0.001
B line	–1.025	0.462	0.448	0.106–0.888	0.027
Cava vein collapse	3.573	0.487	35.635	13.722–92.538	0.001
Mechanical ventilation	–0.803	0.737	0.359	0.106–1.900	0.276
Sepsis	0.584	0.466	1.793	0.719–4.470	0.210

*CI* confidence interval

### P20 The use of renal Doppler ultrasonography in critically ill patients with acute kidney injury

#### Rogerio Passos^1,2^, Erica Melo^1^, Aline Pontara^1^, Octavio Messeder^2^, Augusto Farias^2^, João Gabriel Ramos^1^, Juliana Caldas^1^, Fernanda Coelho^1,2^, Maria Fernanda Coelho^2^

##### ^1^HSR – Hospital São Rafael, Salvador, Bahia, Brazil; ^2^HP – Hospital Português da Bahia, Salvador, Bahia, Brazil

**Background**

Acute kidney injury (AKI) is one of the major organic dysfunctions that occurs in critically ill patients. Ultrasonography of the kidneys and urinary tract is a tool used by nephrologists as a way of evaluating this pathology. Despite this practice, its clinical utility in determining the cause of AKI is poorly established.

**Methods**

This was a prospective cohort study of critically ill patients that underwent renal ultrasound for AKI over a 5-year period at the Hospital Português da Bahia. The renal Resistive Index (RI) was determined by Doppler sonography.

**Results**

Over the 5-year period, 350 renal ultrasounds were performed for the evaluation of AKI. Renal ultrasound was normal in 76% of patients. Hydronephrosis was detected in only 6% of cases and in only 2% of the cases was obstructive uropathy considered the cause of AKI. Less than 1% of patients had urinary tract obstruction on ultrasound without a suggestive medical history. Median renal RIs were 0.68 (0.62–0.75) in patients with sepsis-related AKI and 0.74 (0.72–0.80) in patients with nonsepsis-related AKI (*p* = 0.001). RIs were 0.72 (0.70–0.79) in transient AKI and 0.76 (0.70–0.80) in persistent AKI (*p* = 0.64).

**Conclusions**

The role of renal ultrasonography in critically ill patients with acute renal injury still needs to be determined; in patients with a history suggestive of urinary tract obstruction, renal ultrasound for evaluation of AKI should be indicated. The use of renal RI could be a tool to reflect vasomotor tone in patients with AKI related to sepsis.

### P21 Predictors of mortality in patients undergoing continuous renal replacement therapy

#### Filipe Utuari de Andrade Coelho^1^, Kelly Cristina Marques Galvão^1^, Eduarda Ribeiro dos Santos^1^, Camila Takao Lopes^2^, Beatriz Murata Murakami^1^

##### ^1^HIAE – Hospital Israelita Albert Einstein, São Paulo, SP, Brazil; ^2^UNIFESP – Universidade Federal de São Paulo, São Paulo, SP, Brazil

**Background**

Continuous renal replacement therapy (CRRT) provides gradual fluid removal and solute clearance. It is the modality of renal support in critically ill patients with renal failure who are hemodynamically unstable [1]. Because the mortality rate within 24 h of onset of CRRT can be as high as 68.4%, it is relevant that predictors of mortality are investigated [2].

**Objective**

We aimed to identify predictors of mortality in patients undergoing CRRT.

**Methods**

This was a retrospective cohort study including all adult patients undergoing CRRT in 2016 in an intensive care unit of a general, private hospital in São Paulo, Brazil. The predictors were identified through a stepwise multiple logistic regression model using death as the dependent variable, with significant demographic and clinical variables derived from univariate analysis as covariates, with exclusion of variables to mitigate multicollinearity effects on the results. The study was approved by the Hospital Israelita Albert Einstein Institution’s Ethics Board, approval number 62115616.6.0000.0071.

**Results**

Adult patients (*n* = 123) under intensive care, mainly due to respiratory failure (26.0%), sepsis and septic shock (21.1%), and postoperative recovery (17.8%), were submitted to CRRT for a mean period of 7.9 ± 8.7 days; 64.2% were male and the mean age was 69.7 ± 16.2 years; 62 (50.4%) died. In the univariate analysis, the variables associated with mortality were hypotension (odds ratio (OR) 2.43, 95% confidence interval (CI) 1.12–5.40; *p* = 0.026), use of noradrenaline (OR 14.94, 95% CI 2.80–276.87; *p* = 0.011), use of mechanical ventilation (OR 8.23, 95% CI 2.59–36.74; *p* = 0.001), and length of mechanical ventilation (OR 1.14, 95% CI 1.07–1.23; *p*=0.001).

**Conclusions**

The predictors of mortality in patients undergoing CRRT were use of noradrenaline, cardiorespiratory arrest, and length of mechanical ventilation.

**References**

[1] Tandukar S, Palevsky PM. Continuous renal replacement therapy: who, when, why, and how. Chest 2019;155(3):626–38.

[2] Gonzalez CA, Pinto JL, Orozco V, et al. Early mortality risk factors at the beginning of continuous renal replacement therapy for acute kidney injury. Cogent Med 2018;5(1):1407485.

### P22 Comparison of hemodynamic parameters among continuous, intermittent, and hybrid renal replacement therapy in acute kidney injury in intensive care unit patients: a systematic review of randomized clinical trials

#### Diana Silva Russo^1^, Claudia Severgnini Eugenio^1^, Silvia Rios Vieira^1^, Regis Goulart Rosa^3^, Cassiano Teixeira^3^, Illan George Balestrin^3^, Luis Rovella Junior^3^

##### ^1^UFRGS – Universidade Federal do Rio Grande do Sul, Porto Alegre, RS, Brazil; ^2^UFRGS – Universidade Federal do Rio Grande do Sul, Porto Alegre, RS, Brazil; ^3^HMV – Hospital Moinhos de Vento, Porto Alegre, RS, Brazil

**Background**

The use of renal replacement therapy (RRT) in acute kidney injury (AKI) patients in the intensive care unit (ICU) is associated with high hemodynamic instability, leading to an in-hospital mortality of about 50%. AKI is a frequent complication in the ICU and is independently associated with the development of end-stage renal disease and higher mortality rates [1,2]. Despite the significant advances in medical treatments, AKI requiring RRT in the ICU is associated an increased in-hospital mortality of about 50% [3].

**Objective**

The aim of this study was to compare hemodynamic parameters among continuous, intermittent, and hybrid RRT in AKI in ICU patients.

**Methods**

This was a systematic review conducted in accordance with PRISMA and registered at the PROSPERO database (CRD42018086504). Randomized clinical trials (RCTs) involving patients with AKI in the ICU submitted to continuous, intermittent, or hybrid RRT were included. We investigated the PubMed, Embase and Cochrane databases.

**Results**

Out of 3442 citations retrieved, 12 RCTs were included, representing 1419 patients. Most of the studies did not find differences in hemodynamic parameters across different RTT modalities, except a continuous venovenous hemofiltration (CVVH) group heart rate decrease after 1 and 4 h in comparison with an intermittent hemodialysis (IHD) group, an increase in systolic blood pressure after 0.5 and 2 h of CVVH in contrast to IHD, and significantly higher doses of dobutamine in patients from the continuous venovenous hemodiafiltration (CVVHDF) group when compared with IHD. Lower baseline mean arterial pressure (MAP), greater MAP variation on dialysis, higher number of pressors at baseline, and an increase in pressor dose during dialysis were associated with shorter survival time; greater MAP variation on dialysis was negatively correlated with renal recovery.

**Conclusions**

Most of the studies did not find differences between the groups in relation to heart rate, blood pressure, hypotensive episodes, or use of catecholamines. We believe that this study can provide important insights for further clinical trials designed specifically to evaluate hemodynamic parameters across different types of RRT.

**References**

[1] Ishani A, Xue JL, Himmelfarb J, et al. Acute kidney injury increases risk of ESRD among elderly. J Am Soc Nephrol 2009;20:223–8.

[2] Morgera S, Kraft AK, Siebert G, et al. Long-term outcomes in acute renal failure patients treated with continuous renal replacement therapies. Am J Kidney Dis 2002;40:275–9.

[3] Uchino S, Kellum JA, Bellomo R, et al. Acute renal failure in critically ill patients: a multinational, multicenter study. JAMA 2005;294:813–8.

## Neurology

### P23 Predictors of mortality after subarachnoid hemorrhage: a retrospective multicenter cohort study

#### Pedro Kurtz^1^, Fabio Taccone^2^, Bruno Gonçalves^1^, Marcio Soares^3^, Fernando Bozza^3^, Maristela Machado^4^, Marcelo Maia^5^, Marcus Ferez^6^, Carlos Nassif^7^, Cassia Shinotsuka^1^, Jorge Salluh^3^

##### ^1^IECPN – Instituto Estadual do Cérebro Paulo Niemeyer, Rio de Janeiro, RJ, Brazil; ^2^Free University of Brussels, Brussels, Belgium; ^3^IDOR – D ´Or Institute for Research and Education, Rio de Janeiro, RJ, Brazil; ^4^HAP – Hospital Agenor Paiva, Salvador, BA, Brazil; ^5^Hospital Santa Luzia, Viana do Castelo, Portugal; ^6^HSF – Hospital Sao Francisco, Brazilia, DF, Brazil; ^7^HNJ – Hospital Nove de Julho, São Paulo, SP, Brazil

**Background**

Aneurysmal subarachnoid hemorrhage (SAH) is an acute, and often catastrophic, cerebrovascular event, with high mortality and morbidity. Data on predictors of mortality in low- and middle-income countries are scarce.

**Objective**

The purpose of this study was to investigate clinical predictors of hospital mortality in patients admitted with subarachnoid hemorrhage in a large sample of Brazilian intensive care units (ICUs).

**Methods**

We performed a retrospective cohort study of patients admitted with spontaneous SAH to 57 hospitals in Brazil during 2014 and 2015. We retrieved the clinical and outcome data of patients from an electronic ICU quality registry. Simplified Acute Physiology III (SAPS 3) non-Neuro and Sequential Organ Failure Assessment (SOFA) non-Neuro scores were calculated, subtracting the Glasgow Coma Scale (GCS) contribution from the original score values. We used mixed multivariable logistic regression analysis to identify factors associated with hospital mortality.

**Results**

A total of 1114 patients were included. Fifty-five percent (*n* = 610) of patients were female and 71% were admitted from the emergency room while 18% were transferred from another hospital. Median age was 57 years (interquartile range (IQR) 45–72) and median ICU length of stay was 5 days (IQR 2–10). Median SAPS 3 non-Neuro score was 46 (38–55) and SOFA non-Neuro score was 2 (0–5). A total of 446 (30%) patients presented with poor grade SAH (World Federation of Neurosurgeons grading scale IV and V) and hospital mortality was 35%. In univariate comparisons, nonsurvivors were older and had higher SAPS 3 non-Neuro and SOFA non-Neuro scores (all (all *P*<0.001). Poor grade SAH, use of vasopressors, mechanical ventilation, intracranial pressure monitoring, external ventricular drainage, blood transfusions and renal replacement therapy were all more frequent among nonsurvivors (all P<0.001). Mortality was also higher with initial lactate above 2 mmol/L, in those admitted to public hospitals and when admission to ICU was delayed more than 24 hours after ictus. After adjusting for

common predictors (age, gender and WFNS) SAPS 3 non-Neuro, SOFA non-Neuro, early vasopressor use and admission to a public hospital were independently associated with hospital mortality. Moreover, the area under the curve for prediction of mortality with SAPS3, SOFA and WFNS was 0.86.

**Conclusions**

Mortality is elevated and highly variable in this large sample of SAH patients. Age and severity of clinical presentation, both systemic and neurological, as well as the presence of organ dysfunction were associated with mortality.

**Fig. 1 (Abstract P23).**
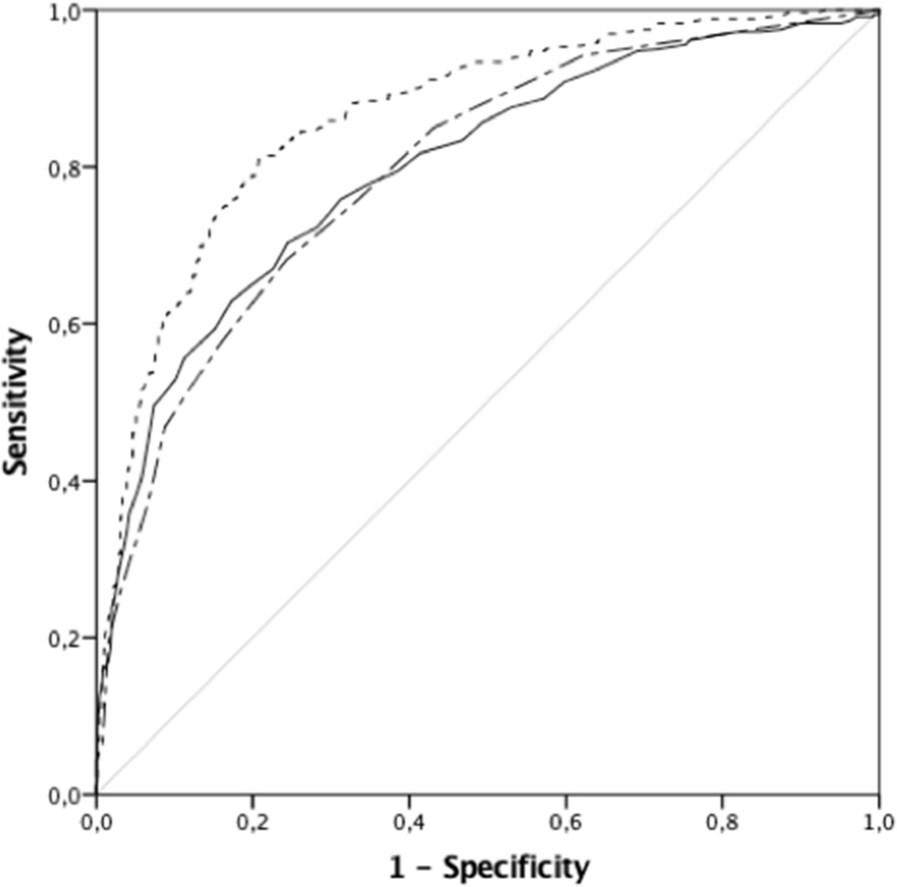
Area under the receiver operating characteristic curve. Simplified Acute Physiology III (SAPS 3) non-Neuro, –0.8; Sequential Organ Failure Assessment (SOFA) non-Neuro, –0.79; SAPS 3 non-Neuro SOFA non-Neuro WFNS –0.86

### P24 Clinical predictors of early and late mortality after cardiac arrest: a retrospective multicenter cohort study

#### Pedro Kurtz^1^, Christian Storm^2^, Marcio Soares^3^, Fernando Bozza^3^, Ulisses Melo^4^, Dieter Araya^5^, Bruno Mazza^6^, Marcelo Santino^7^, Jorge Salluh^3^

##### ^1^IECPN – Paulo Niemeyer State Brain Institute, Rio de Janeiro, RJ, Brazil; ^2^Charité – Universitätsmedizin, Berlin, Germany; ^3^IDOR – D ´Or Institute for Research and Education, Rio de Janeiro, RJ, Brazil; ^4^HEAT – Hospital Estadual Alberto Torres, São Gonçalo, RJ, Brazil; ^5^HSP – Hospital Santa Paula, São Paulo, SP, Brazil; ^6^HS – Hospital Samaritano, São Paulo, SP, Brazil; ^7^HBD – Hospital Barra D’Or, Rio de Janeiro, RJ, Brazil

**Background**

Data on the outcome of survivors of cardiac arrest patients in low- and middle-income countries is scarce.

**Objective**

The purpose of this study was to investigate clinical predictors of hospital mortality in patients resuscitated after cardiac arrest in a large sample of Brazilian intensive care units (ICUs).

**Methods**

We performed a retrospective cohort study of survivors from cardiac arrest in 57 hospitals in Brazil during 2014 and 2015. We retrieved clinical and outcome data of patients from an electronic ICU quality registry. We used multivariable logistic regression analysis to identify factors associated with hospital mortality.

**Results**

A total of 2295 patients were included. Both patients with primary admission diagnosis of cardiac arrest (*n* = 800, 35%) and those that arrested on admission to the ICU (65%) were included. Median age was 67 years (interquartile range (IQR) 54–79) and 53% were male. Median Simplified Acute Physiology III score (SAPS 3) was 70 (57–83), Sequential Organ Failure Assessment (SOFA) score was 10 (7–13) and hospital mortality was 83%. Among nonsurvivors, 47% died in the first 48 h of ICU admission (early mortality). Only 1% received therapeutic hypothermia and 6% underwent withholding/withdrawal of life support. After adjusting for SAPS 3 and SOFA, early mortality was associated with hemodynamic compromise (systolic blood pressure 4 mmol/l and use of vasopressors) and hypo- or hyperthermia. Late mortality was independently associated with delayed admission to the ICU (>48 h) and palliative care.

**Conclusions**

This large cohort of post-cardiac arrest survivors demonstrates extremely high mortality rates and negligible implementation of hypothermia in Brazil, with half of nonsurvivors dying within 48 h of admission with severe hemodynamic compromise and organ dysfunction. Late mortality was related to delayed admission to ICU and withdrawal/withholding measures.

### P25 Mental illness among survivors of critical care: a multicenter prospective cohort study

#### Regis Rosa, Caroline Robinson, Renata Kochhmann, Evelin Sanchez, Daniel Schneider, Rodrigo Jeffman, Debora Mariani, Denise de Souza, Rosa dos Santos, Gabriela Rech, Cassiano Teixeira

##### HMV – Hospital Moinhos de Vento, Porto Alegre, RS, Brazil

**Background**

As more patients are surviving intensive care, mental health concerns in survivors have become a research priority. Among these, anxiety, depression and post-traumatic stress disorder (PTSD) may have an important impact on the quality of life of critical care survivors. However, data on the mental illness burden in this specific population are conflicting.

**Objective**

We aimed to identify the prevalence and predictors of mental health disability among general adult patients who were discharged alive from intensive care units (ICUs).

**Methods**

This was a multicenter prospective cohort study performed in 10 tertiary hospitals in Brazil from May 2014 to December 2018. Adult ICU survivors with an ICU stay >72 h for medical and emergency surgical admissions or >120 h for elective surgical admissions were followed up for 6 months. The main outcomes were anxiety and depression symptoms assessed by the Hospital Anxiety and Depression Scale (HADS) , PTSD symptoms assessed by the Impact Event Scale-6 (IES-6), and quality of life assessed by the short-form (SF)-12 questionnaire.

**Results**

A total of 1616 post-ICU patients were enrolled. Of these, 579 were assessed for mental health symptoms at 6 months. The prevalence of any mental health disability was 36.3% (210 patients). The prevalence of anxiety, depression, and PTSD were 24.2% (140 patients), 20.9% (121 patients), and 15.9% (89 patients), respectively. Patients with post-ICU mental health disabilities had lower scores of quality of life in both physical and mental domains compared with patients without post-ICU mental health disabilities. Age <65 years (p<0.001), female sex (p=0.01), history of depression (p<0.001), need of renal replacement therapy (p=0.02) and decrease in functional status (p<0.001) were independently associated with anxiety. History of depression (p<0.001), increased risk of death at ICU admission (p=0.04) and decrease in functional status (p=0.003) were independently associated with depression. Age <65 years (p=0.01) and decrease in functional status (p=0.001) were independently associated with PTSD.

**Conclusions**

The high burden of mental health disability among survivors of critical care is a matter of concern for public health due to its negative impact on quality of life. The network of potential risk factors for anxiety, depression, and PTSD among post-ICU patients is complex and involves factors of multiple domains such as sociodemographic, pre-morbid health, and ICU-related variables and sequela of critical illness.

### P26 Transfusion requirements after head trauma (TRAHT): a randomized clinical trial

#### André Gobatto^1,2^, Milena Link^1^, Davi Solla^1^, Estevão Bassi^1^, Paulo Tierno^1^, Wellingson Paiva^1^, Fabio Taccone^3^, Luiz Marcelo Malbouisson^1^

##### ^1^USP – Universidade de São Paulo, São Paulo, SP, Brazil; ^2^HSR – Hospital São Rafael, Salvador, Bahia, Brazil; ^3^Erasme Hospital, Brussels, Belgium)

**Background**

Anemia is frequent among patients with traumatic brain injury (TBI) and is associated with an increased risk of poor outcome. The optimal hemoglobin concentration to trigger red blood cell (RBC) transfusion in TBI patients is not clearly defined.

**Objective**

We aimed to evaluate the feasibility and safety of two different hemoglobin thresholds for blood transfusion in patients with moderate or severe TBI.

**Methods**

All eligible consecutive adult patients admitted to the intensive care unit (ICU) with moderate or severe TBI were randomized to a ‘restrictive’ (hemoglobin transfusion threshold of 7 g/dl) or a ‘liberal’ (threshold 9 g/dl) transfusion strategy. The transfusion strategy was continued for up to 14 days or until ICU discharge. The primary outcome was the mean hemoglobin difference between groups. Secondary outcomes included transfusion requirements, intracranial pressure management, cerebral hemodynamics, length of stay, mortality, and 6-month neurological outcome.

**Results**

A total of 44 patients were randomized, 21 patients to the liberal group and 23 to the restrictive group. There were no baseline differences between the groups. The mean hemoglobin concentrations over the 14-day period were 8.4 ± 1.0 and 9.3 ± 1.3 (p<0.01) in the restrictive and liberal groups, respectively. Fewer RBC units were administered in the restrictive than in the liberal group (35 vs. 66, p=0.02). There was a negative correlation (r=-0.265, p<0.01) between hemoglobin concentration and middle cerebral artery flow velocity as evaluated by transcranial Doppler and the incidence of post-traumatic vasospasm was significantly lower in the liberal strategy group (4/21, 3% vs. 15/23, 65%; p<0.01). Hospital mortality was higher in the restrictive than in the liberal group (7/23 vs. 1/21; p=0.048) and the liberal group tended to have a better neurological status at 6 months (p = 0.06).

**Conclusions**

The trial reached feasibility criteria. The restrictive group had lower hemoglobin concentrations and received fewer RBC transfusions. Hospital mortality was lower and neurological status at 6 months favored the liberal group.

**References**

[1] Lelubre C, Bouzat P, Crippa IA, Taccone FS. Anemia management after acute brain injury. Crit Care 2016;20(1):152.

[2] Robertson CS, Hannay HJ, Yamal JM, Gopinath S, Goodman JC, Tilley BC, et al. Effect of erythropoietin and transfusion threshold on neurological recovery after traumatic brain injury: a randomized clinical trial. JAMA 2014;312(1):36–47.

**Fig. 1 (Abstract P26).**
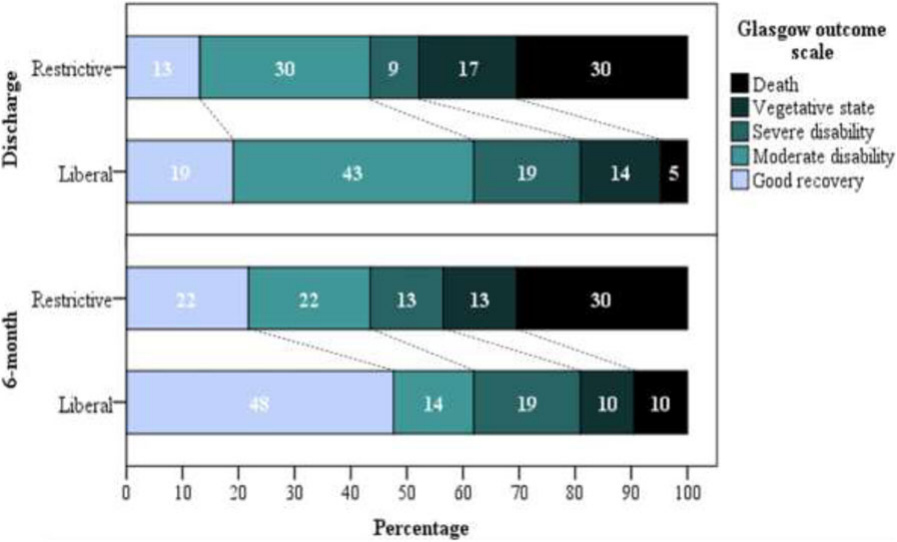
Mean daily hemoglobin concentrations in the liberal and restrictive strategy groups during the first 14 days after intensive care unit admission. ADM admission

**Fig. 2 (Abstract P26).**
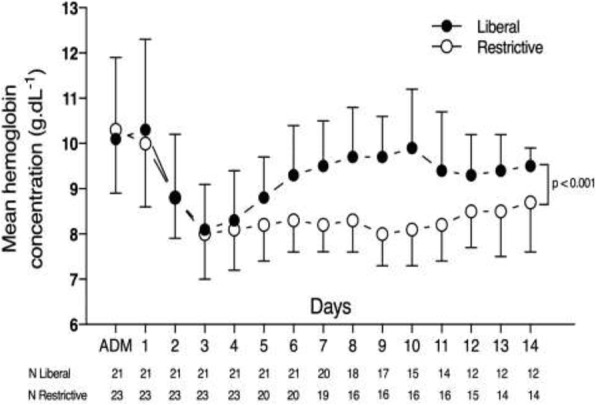
Neurological status at hospital discharge and at 6 months after hospital discharge as measured by the Glasgow Outcome Scale

## Nutrition/Metabolism

### P27 Impact of telemedicine on the quality assurance processes of nutritional therapy in critical patients: multiprofessional nutrition therapy team without borders

#### Glaucia Amaral Santana, Tatiana Scacchetti, Diogo Oliveira Toledo, Evandro Jose de Almeida Figueiredo, João Manoel Da Silva Junior

##### SBIBAE – Sociedade Beneficente Israelita Brazileira Albert Einstein, São Paulo, SP, Brazil

**Background**

The prevalence of malnutrition in hospitalized patients is high and may even increase during hospital stay. Adequate nutritional management can improve this scenario, and, currently, the Multiprofessional Nutrition Therapy Team is the most important instrument in this context for obtaining improvement. However, a Multiprofessional Nutrition Therapy Team is not always available in hospitals and the alternative would be to use the telemedicine service to increase the access and reach of specialized professionals.

**Objective**

This study aims to compare the care assistance quality of nutritional therapy in critical patients, analyzing indicators before and after the implementation of the telemedicine service in a tertiary hospital.

**Methods**

This is a case–control study comparing the indicators of the proportion of the prescribed volume that was infused (volume adequacy), the proportion of the protein target reached, and the diarrhea rate prior to the implementation of telemedicine first period (October 2016 to February 2017) and after the telemedicine second period (from June 2017 to October 2017). All patients who received enteral nutritional therapy during the period participated in the study.

**Results**

A total of 150 patients (65 patients from first period and 85 from the second period) participated in the study. The median age was 57.5 (interquartile range (IQR) 44–64) years, 51.8% were male and 48.2% were female, and most of the patients were receiving treatment for pulmonary focus sepsis. In general, the median percentage of diet volume infused was 88.8% (75.7–98.1%), the patients received a median of 1.13 (0.9–1.33) g/kg of protein, and 14.7% presented with diarrhea. The crude diet infused volume increased from 83.3% (IQR 71.3–95.7%) in the first period to 91.7% (IRQ 80.1–99.2%) in the second period (*p* = 0.01) and the same has occurred for the protein received, but this was not statistically significant (1.08 ± 0.3 g/kg in the first period and 1.16 ± 0.3 g/kg in the second period; *p* = 0.10). After adjusting for age, patients enrolled in the first period (19.6%, 95% confidence interval (CI) 9.4–36.1) compared with those enrolled in second period (0.0%, 95% CI 0.0–7.2) exhibited a lower risk of diarrhea (odds ratio (OR) 0.04, 95% CI 0.02–0.7; *p* = 0.03).

**Conclusions**

The telemedicine seems to achieve good results in the follow-up of patients who received enteral nutrition when compared with no specialized professional follow-up; this study demonstrated that it improved the care quality indicators.

**References**

[1] Bussab WdO, Morettin PA. Estatística básica, 6ª ed. São Paulo: Saraiva; 2010.

[2] Neter J, Kutner MH, Nachtsheim CJ, Wasserman W. Applied linear statistical models. Chicago: Irwin; 1996.

[3] Hosmer Jr DW, S. Lemeshow S. Applied logistic regression. John Wiley & Sons; 2004.

[4] Faraway JJ. Extending the linear model with R: generalized linear, mixed effects and nonparametric regression models. Boca Raton, FL: Chapman & Hall/CRC; 2006.

[5] R Core Team. R: a language and environment for statistical computing, Vienna: R Foundation for Statistical Computing; 2015.

[6] PASS 14 Power Analysis and Sample Size Software. NCSS, LLC, Kaysville, Utah, USA; 2015.

## Safety/Quality/Management

### P28 Dysphagia in adult patients: a Brazilian simple screen validated against videofluoroscopy

#### Maíra Santilli de Lima^1^, Fernanda Chiarion Sassi^2^, Gisele Chagas de Medeiros^1^, Shri Krishna Jayanthi^3^, Claudia Regina Furquim de Andrade^2^

##### ^1^HCFMUSP – Divisão de Fonoaudiologia do Hospital das Clínicas da FMUSP, São Paulo, SP, Brazil; ^2^FOFITO – FMUSP - Departamento de Fono, Fisio e TO da FMUSP, São Paulo, SP, Brazil; ^3^InrRad HCFMUSP – Instituto de Radiologia do Hospital das Clínicas da FMUSP, São Paulo, SP, Brazil

**Background**

Dysphagia has already been reported to increase the length of hospital stay and the risk of mortality in all age groups [1]. A simple swallowing screen can be used to identify patients at risk for bronchopulmonary penetration/aspiration, the need for further swallowing assessment, and the safety of patient oral intake [2]. Currently, no consensus exists on a standard method of clinical swallowing assessment [3].

**Objective**

The purpose of the present study was to assess the validity of a simple screening swallowing instrument used in a large public-school hospital in Brazil.

**Methods**

The study was approved by the Ethics Committee for the Analysis of Research Projects of Hospital das Clínicas School of Medicine University of São Paulo, approval number 1.781.177. The Dysphagia Risk Evaluation Protocol (DREP) screening version contains four items (altered cervical auscultation, altered vocal quality, coughing, and choking after the swallow) that were previously indicated as independent risk factors associated with the presence of dysphagia. Trained speech-language pathologists administered and scored the DREP screening version to consecutive patients referred by the hospital’s medical team to perform a videofluoroscopy of swallowing (VFSS) Twenty percent of all enrolled patients were then randomly allocated to VFSS 24 h after being submitted to the DREP screening version.

**Results**

Two hundred and eleven patients received the swallowing screen: 99 failed and 112 passed. One in every five patients were randomized to receive a VFSS (Fig. 1). The DREP screening version demonstrated excellent validity with sensitivity of 92.9%, specificity 75.0%, negative predictive values of 95.5%, and an accuracy of 80.9% (Table 1).

**Conclusions**

The DREP screening version is a simple and accurate tool to identify bronchopulmonary penetration/aspiration in patients who are not tube fed, who have good level of alertness, no history of repetitive pneumonia or current respiratory compromise, and are not using a tracheostomy tube.

**References**

[1] Altman KW, Yu GP, Schaefer SD. Consequence of dysphagia in hospitalized patient. Arch Otolaryngol 2010;136:784–9.

[2] Hinchey JA, Shephard T, Furie K, Smith D, Wang D, Tonn S. Formal dysphagia screening protocols prevent pneumonia. Stroke 2005;36:1972–6.

[3] Fedder WN. Review of evidence-based nursing protocols for dysphagia assessment. Stroke 2017;48:99–101.

**Fig. 1 (Abstract P28).**
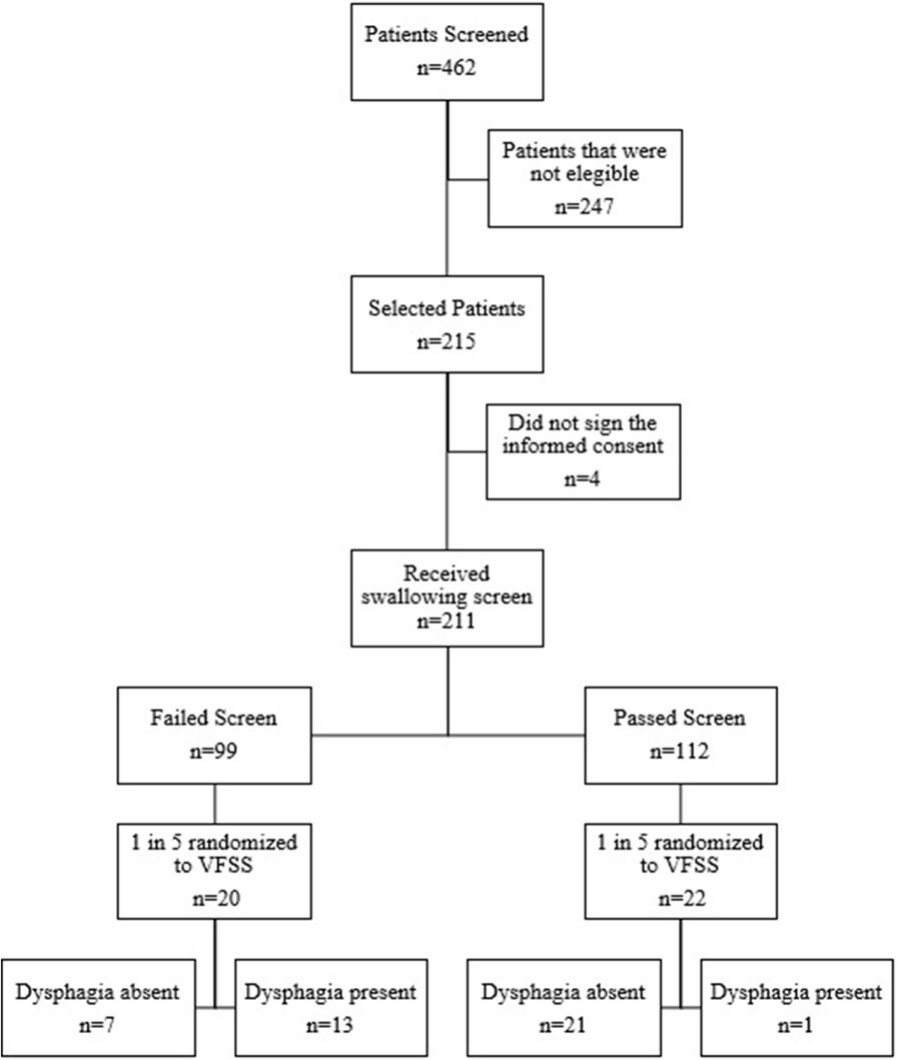
Flow diagram for the DREP validation. VFSS videofluoroscopy of swallowing

**Table 1 (Abstract P28).**
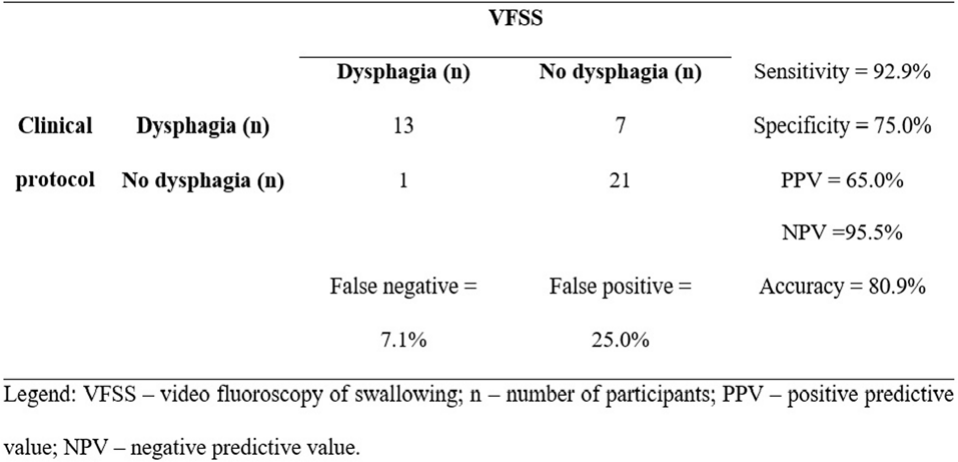
Comparison of the accuracy measures of the clinical and VFSS results

### P29 Antimicrobials in the intensive care unit: do we prescribe safely?

#### Ana FGA Pinheiro, Carlos AR Feijó

##### HGF – Hospital Geral de Fortaleza, Fortaleza, Ceará, Brazil

**Background**

Worldwide, patients are subject to medication errors due to poorly written or misinterpreted prescriptions, which can lead to adverse events ranging from those that are mild to even death. To minimize this type of error, it is necessary to use strategies to avoid their occurrence such as simple guidelines to prescribing physicians and/or the use of electronic prescriptions.

**Objective**

We aimed to evaluate the quality of intravenous antimicrobial prescriptions in an intensive care unit (ICU) of a quaternary hospital in Fortaleza, Ceara, Brazil, during a period of 1 month.

**Methods**

This is a descriptive, observational, cross-sectional and retrospective study. We identified all the antimicrobials for intravenous use prescribed during the month of November 2018 and analyzed the presence of the following items: 1) generic name; 2) concentration; 3) pharmaceutical form; 4) dose; 5) volume; 6) diluent; 7) administration route; 8) infusion rate; and 9) dosage. We considered any prescription that contained all nine items as safe.

**Results**

Three hundred and ninety-four antimicrobial prescriptions were identified in the period. Of these, we found adhesion rates of 94.9% for generic name, 26.7% for concentration, 25.9% for pharmaceutical form, 100% for dose, 90.9% for volume, 91.4% for diluent, 99.2 % for administration route, 68.3% for infusion rate, and 98.2% for dosage. Of the total, only 19.5% contained all the recommended items.

**Conclusions**

Our data suggest that we did not prescribe antimicrobials safely in the unit since only one in five prescriptions were complete. It is necessary to develop counseling strategies for physicians so that low adherence to the full prescription, an indirect indicator of low quality, does not become an error and lead to adverse events in patients.

**Reference**

[1] Marchete AGG, Martins BA, Corti GS, Beijamini V. Evaluation of antimicrobial prescriptions to pediatric patients in a Northern Espirito Santos's Hospital. Rev Bras Farm 2010;91(4):176–82.

### P30 Analysis of an intervention on antimicrobial prescriptions in an intensive care unit in Fortaleza, Ceará, Brazil

#### Ana FGA Pinheiro, Carlos AR Feijó

##### HGF – Hospital Geral de Fortaleza, Fortaleza, Ceará, Brazil

**Background**

With the evolution of the therapeutic arsenal, as well as a growing concern on patient safety, lots of information has become necessary on a medical prescription to ensure its correct understanding. This information, when absent, incomplete or illegible, adds a high probability of occurrence of errors, sometimes fatal errors. The investigation of prescribing habits allows us to detect such failures, which makes possible the implementation of appropriate and timely interventions.

**Objective**

We aimed to analyze the results of intervention on the intravenous antimicrobial prescriptions in an intensive care unit (ICU) of a quaternary hospital in Fortaleza, Ceará, Brazil, during a period of 1 month.

**Methods**

This is an analytical, interventional, before-and-after, longitudinal and retrospective study. We identified all the antimicrobials for intravenous use prescribed during the month of November 2018 and analyzed the presence of the following items: 1) generic name; 2) concentration; 3) pharmaceutical form; 4) dose; 5) volume; 6) diluent; 7) administration route; 8) infusion rate; and 9) dosage. During the month of December, through cell phone messages and printed information, we disclosed to ICU physicians the correct way to prescribe antimicrobials. After that, we collected data from the prescriptions of January 2019 and analyzed the efficiency of the intervention.

**Results**

Three hundred and ninety-four antimicrobial prescriptions were analyzed in November 2018. Of these, 26.7% contained the concentration, 25.9% had the pharmaceutical form, and only 19.5% were complete. In January 2019, 428 antimicrobial prescriptions were analyzed. We observed an increase in the concentration prescription (46.3%) and in the pharmaceutical form (45.1%), with a statistically significant difference (p <0.05). In addition, 42.8% of the prescriptions had all the recommended items, which represented an increase of 119.5% compared to the previous period, also with statistical significance (p <0.05). Despite this, adherence to the infusion rate prescription remained low [68.3% (November) vs 63.1% (January)]. The remaining items were present in more than 90% of the prescriptions in the two analyzed months.

**Conclusions**

Our study suggests that the intervention can be considered an efficient measure, but with results still lower than those expected of a safe antimicrobial prescription. It is therefore necessary that measures are taken to ensure the presence of essential information for a quality prescription, mainly through permanent orientation with the physicians.

**Reference**

[1] Marchete AGG, Martins BA, Corti GS, Beijamini V. Evaluation of antimicrobial prescriptions to pediatric patients in a Northern Espirito Santos's Hospital. Rev Bras Farm 2010;91(4):176–82.

## Sepsis/Septic Shock

### P31 Meropenem extended infusion to guarantee drug effectiveness against nosocomial MIC 4 mg/l strains in burn patients at the earlier period of septic shock

#### Léonard De Vinci Kanda Kupa^1^, João Manoel da Silva Junior^2^, Elson Mendes Silvia Junior^2^, Amanda Maria Ribas Rosa de Oliveira^2^, Aline Sandre Gomides^2^, Cristina Carvalho da Silva^2^, Gabriela Aparecida Ferreira^2^, Thiago Camara Oliveira^2^, Nilo José Coelho Duarte^3^, David de Souza Gomez^2^, Camila Salinas Guillaux^1^, Silvia Regina Cavani Jorge Santos^1^

##### ^1^PC – Pharmacokinetics Center, FCF USP, São Paulo, SP, Brazil; ^2^DCPQ – Division of Plastic Surgery and Burns, ICHC-FMUSP, São Paulo, SP, Brazil; ^3^DLC – Division of Central Laboratory, HCFMUSP, São Paulo, SP, Brazil

**Background**

The meropenem recommended dose usually cannot achieve its target in critically ill septic patients in the intensive care unit (ICU) against the most common MIC >2 mg/l strains that can impact the desired outcome [1].

**Objective**

The aim of the study was to investigate meropenem 3 g daily by extended infusion based on the pharmacokinetic-pharmacodynamic (PK/PD) approach in burn patients receiving the initial dose regimen against nosocomial-susceptible strains.

**Methods**

Ethical approval was obtained (no. 069/09-2015). Thirteen burn patients were investigated. They had a median age of 27 years, weight of 70 kg, with 34% median total burn surface area, and Simplified Acute Physiology III score (SAPS 3) of 58; 8/13 had inhalation injury, with preserved renal function undergoing meropenem treatment at the earlier period of septic shock receiving 1 g every 8 h as a 3-h infusion. A series of two blood samples were collected (1.5 ml each) at the steady state for drug serum measurement by liquid chromatography [2]. PK results from burn patients were compared with data previously described in healthy volunteers [3]. The PK/PD approach was performed to estimate the probability of target attainment (PTA) based on the predictive index of drug effectiveness (100% fT > MIC) [4].

**Results**

Effective free serum trough levels occurred at the recommended dose regimen (1 g q8h 3-h infusion; Fig. 1A and B). Total isolates of bacterial infection were stratified in *Enterobacteriaceae* and *Pseudomonas aeruginosa* at the earlier period of septic shock (Fig. 1C–E). Target was attained for all patients by eradication of nosocomial pathogens up to MIC 4 mg/l (Fig. 1F). Pharmacokinetics are illustrated in Fig. 2.

**Conclusions**

The meropenem serum level is altered in the earlier period of septic shock in burn patients with impacts on PK and drug effectiveness. The desired outcome was reached during the therapy and the clinical cure occurred in all patients against MIC 4 mg/l.

**References**

[1] Gonçalves-Pereira J, Silva NE, Mateus A, et al. Assessment of pharmacokinetic changes of meropenem during therapy in septic critically ill patients. BMC Pharmacol Toxicol 2014;15:21.

[2] Santos et al. J Clin Pharmacol. 2011; 51(9): 1331-32.

[3] Jaruratanasirikul S, Sriwiriyajan S. Comparison of the pharmacodynamics of meropenem in healthy volunteers following administration by intermittent infusion or bolus injection. J Antimicrob Chemother 2003;52(3):518–21.

[4] Calier M, Stove V, Wallis SC, et al. Assays for therapeutic drug monitoring of β-lactam antibiotics: a structured review. Int J Antimicrob Agents 2015;46:367–75.

**Fig. 1 (Abstract P31).**
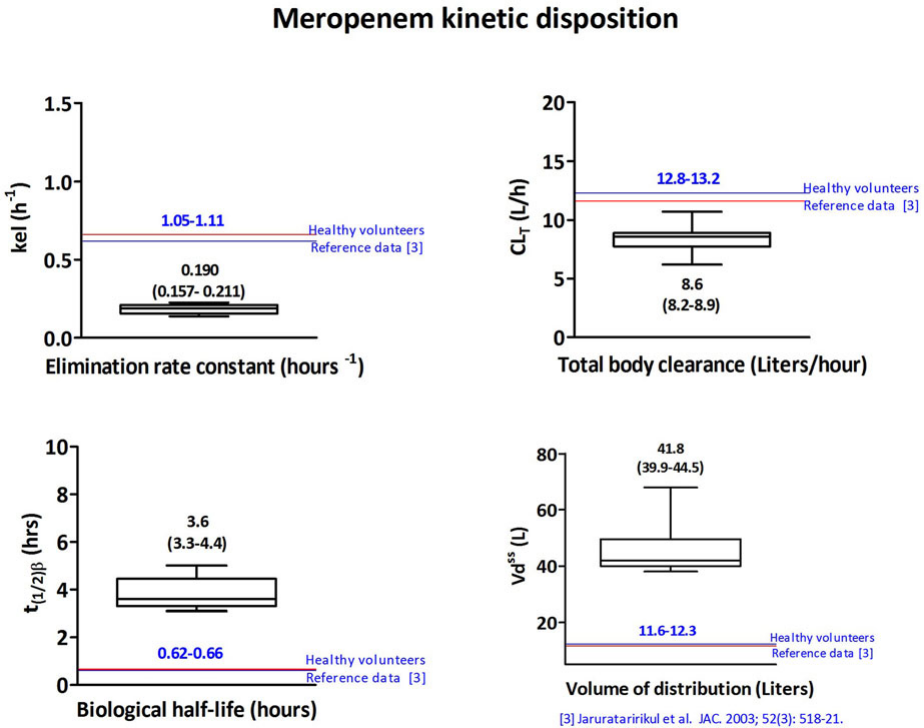
Meropenem therapy in septic burn patients and target attainment. Values are shown as medians (quartiles)

**Fig. 2 (Abstract P31).**
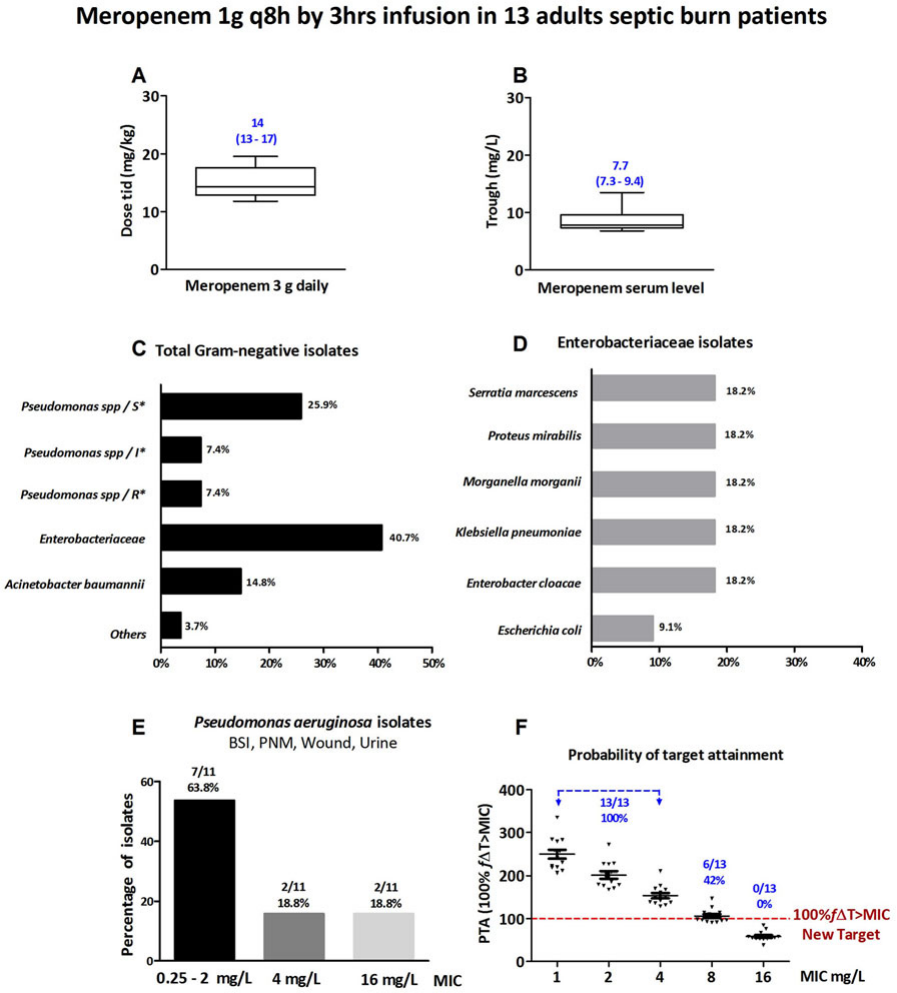
Pharmacokinetics

### P32 Vancomycin dose adjustment in critically ill burn patients using the pharmacokinetic/pharmacodynamic approach against Gram-positive MIC 2 mg/l strains

#### Léonard De Vinci Kanda Kupa^1^, João Manoel da Silva Junior^2^, Elson Mendes Silva Junior^2^, Amanda Maria Ribas Rosa de Oliveira^2^, Aline Sandre Gomides^2^, Cristina Carvalho da Silva^2^, Gabriela Aparecida Ferreira^2^, Thiago Camara Oliveira^2^, Nilo José Coelho Duarte^3^, David de Souza Gomez^2^, Silvia Regina Cavani Jorge Santos^1^

##### ^1^PC – Pharmacokinetics Center, FCF USP, São Paulo, SP, Brazil; ^2^DCPQ – Division of Plastic Surgery and Burns, ICHC-FMUSP, São Paulo, SP, Brazil; ^3^DLC – Division of Central Laboratory, HCFMUSP, São Paulo, SP, Brazil

**Background**

The vancomycin empiric dose regimen usually recommended cannot reach the target in critically ill septic patients in the intensive care unit (ICU) against the most common MIC >1 mg/l strains and this can impact the desired outcome.

**Objective**

The aim of the study was to compare vancomycin 2 g versus 3 g daily based on the pharmacokinetic/pharmacodynamic (PK/PD) approach in ICU burn patients.

**Methods**

Ethical approval was obtained (no. 069/09-2015). Ten burn patients (1 female and nine male) had a median age of 25 years, weight of 78 kg, with 29% total burn surface area, and Simplified Acute Physiology III score (SAPS 3) of 63; 8/10 had an inhalation injury, with preserved renal function undergoing vancomycin therapy (1-h infusion) at the earlier period of septic shock receiving 1 g every 12 h (set 1) or 1 g every 8 h (set 2). A series of two blood samples were collected (1.5 ml each) at the steady state for drug serum measurement by immunoassay. PK results obtained from burn patients were compared with data previously described in healthy volunteers [1]. The PK/PD approach was performed to estimate the probability of target attainment (PTA) based on the predictive index of drug effectiveness (AUC/MIC >400) [2].

**Results**

We demonstrated a significant difference (p<0.0002) between the empiric and adjusted daily dose, figure 1A. Total isolates of gram-positive strains were stratified in *Staphylococus* spp, *Streptococcus* spp and *Enterococcus* faecalis isolated from fluids at the earlier period of septic shock, figure 1B.Target was attained after dose adjustment in 70% (7/10) patients by eradication of nosocomial pathogens up to MIC 2 mg/L, figure 1C. Pharmacokinetics was altered at the earlier period of septic shock, figure 2.

**Conclusions**

Vancomycin serum levels were reduced during septic shock as a consequence of increases in total body clearance and reduction in the biological half-life in burn patients with an impact on drug effectiveness. The desired outcome was reached during the therapy once the clinical cure occurred in 7/10 patients against MIC 2 mg/l.

**References**

[1] Boeckh M, Lode H, Borner K, et al. Pharmacokinetics and serum bactericidal activity of vancomycin alone and in combination with ceftazidime in healthy volunteers. Antimicrob Agents Chemother 1988;32(1):92–5.

[2] Revilla N, Martin-Suarez A, Perez MP, et al. Vancomycin dosing assessment in intensive care unit patients based on a population pharmacokinetic/pharmacodynamic simulation. Br J Clin Pharmacol 2010;70(2):201–12.

**Fig. 1 (Abstract P32).**
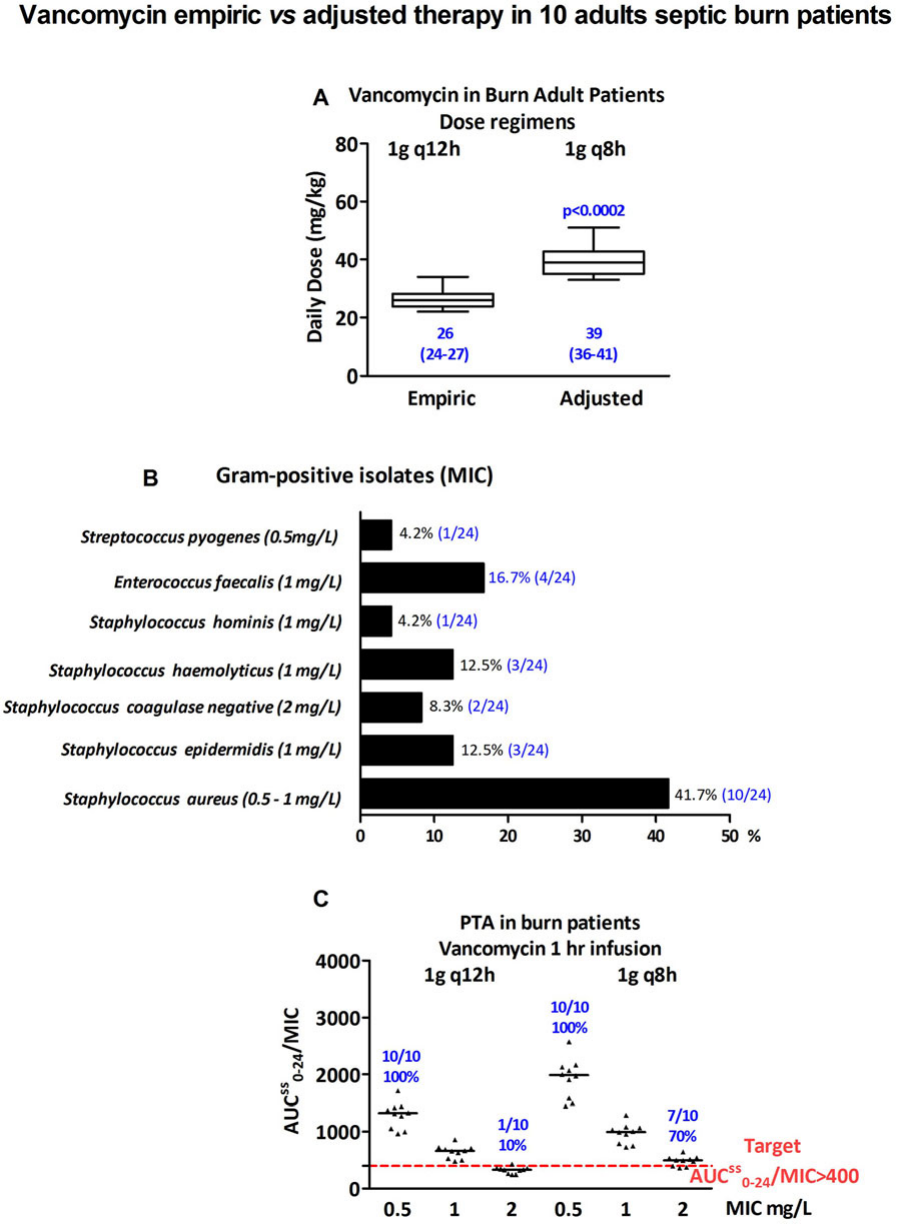
Vancomycin therapy in septic burn adult patients. Values are shown as medians (quartiles)

**Fig. 2 (Abstract P32).**
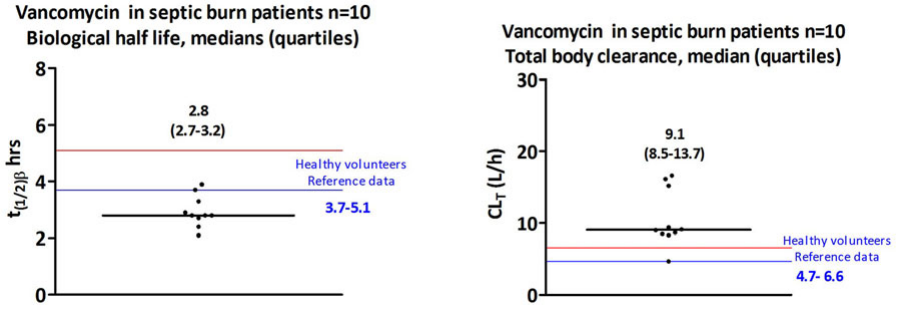
Vancomycin kinetic disposition

### P33 The pharmacokinetic/pharmacodynamic approach in pediatric septic burn patients to guarantee vancomycin effectiveness against *Staphylococcus*.

#### Edvaldo Vieira de Campos^2^, Léonard De Vinci Kanda Kupa^1^, Vedilaine Aparecida Bueno da Silva Macedo^1^, Thaís Vieira de Camargo^1^, João Manoel da Silva Junior^2^, Cristina Carvalho da Silva^2^, José Gomes Pereira Junior^2^, Juliana Gadelha Fedeli^2^, Nilo José Coelho Duarte^3^, Silvia Regina Cavani Jorge Santos^1^, David de Souza Gomez^2^

##### ^1^PC – Pharmacokinetics Center, FCF USP, São Paulo, SP, Brazil; ^2^DCPQ – Division of Plastic Surgery and Burns, ICHC-FMUSP, São Paulo, SP, Brazil; ^3^DLC – Division of Central Laboratory, HCFMUSP, São Paulo, SP, Brazil

**Background**

In septic pediatric burn patients, it is a challenge to maintain effective vancomycin plasma levels, once changes occur in the pharmacokinetics; the control of infection is therefore a challenge in these critically ill pediatric patients [1].

**Objective**

The aim of the study was to investigate vancomycin effectiveness against infections mainly caused by *Staphylococcus* spp. MIC 0.5–2 mg/l in pediatric septic burn patients after the empiric dose (set 1) against the individualized dose regimen (set 2) by a pharmacokinetics/pharmacodynamics (PK/PD) approach.

**Methods**

The protocol was approved (no. 0069/15). Twenty patients (14 male and six female) were investigated, with the following characteristics: creatinine clearance (CrCl) median 241 (quartiles 202–295) ml/min, age 5.0 (3.0–9.5) years, weight 22 (16–38) kg, total burn surface area 30 (20–39) %. Fire was the reason for the burns, inhalation injury occurred in 12 patients, and vasoactive drugs and mechanical ventilation were required in 13/20. Blood was sampled at 3 h and 5 h (1.5 mL each) after the beginning of 1-h infusion. Patients were investigated in two sets after the recommended empiric daily dose (set 1) and after individualized drug therapy (set 2). Drug serum measurements were performed by liquid chromatography. A PK/PD approach was performed based on the predictive index AUC/MIC >400, where MIC is the minimum inhibitory concentration value for each isolated pathogen.

**Results**

We showed a significant difference between the recommended dose regimen and the adjusted dose (Fig. 1A and B). Total isolates related to the infection caused by Gram-positive strains at the earlier period of septic shock are shown in Fig. 1C and D. The percentage of target attainment (PTA) is shown after both therapies in Fig. 1E.

**Conclusions**

The vancomycin serum level is altered at the earlier period of septic shock in pediatric burn patients with an impact on drug effectiveness. After dose adjustment, the desired outcome was obtained for all patients against *S. aureus* MIC 0.5–1 mg/l while the PTA was reduced to 65% (13/20) of patients against MIC 2 mg/l strains.

**Reference**

[1] Gomez DS, et al. Individualized vancomycin dose for paediatric burn patients to achieve PK/PD targets. Burns 2013;39(3):445–50.

**Fig. 1 (Abstract P33).**
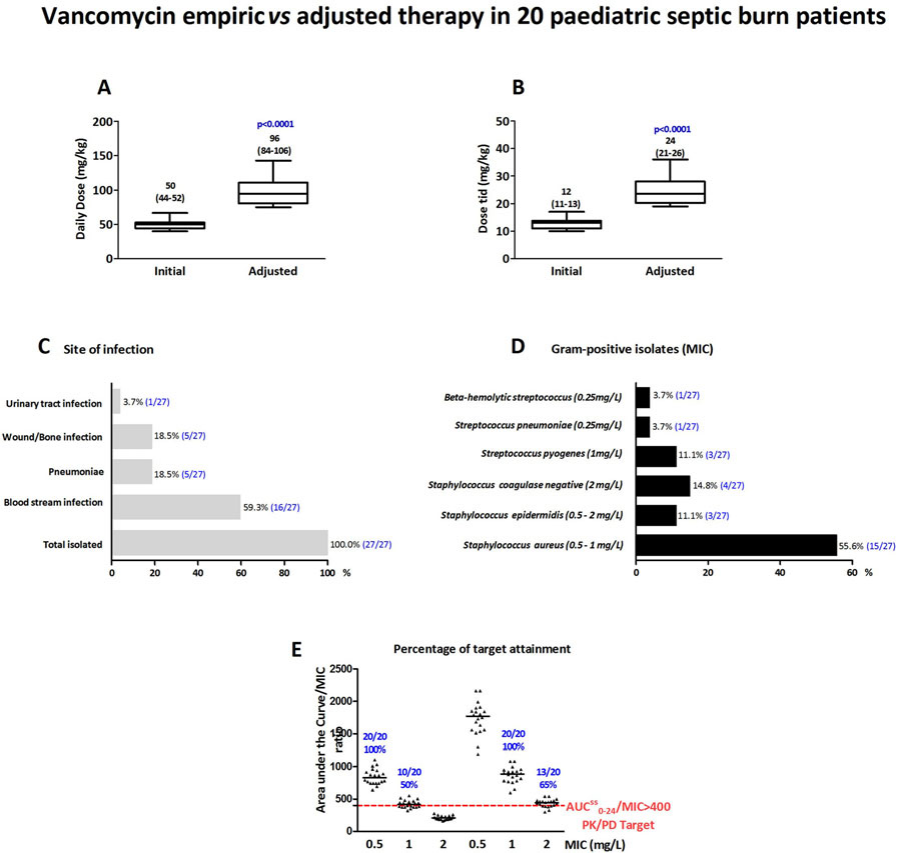
Vancomycin therapy in septic burn pediatric patients. Values are shown as medians (quartiles)

### P34 Vancomycin effectiveness in critically septic pediatric burn patients compared with nonburn patients requires dose adjustment for target attainment against *Staphylococcus* spp. MIC 1 mg/l

#### Frederico Ribeiro Pires^1^, Artur Figueiredo Delgado^1^, Edvaldo Vieira de Campos^2^, Cristina Carvalho da Silva^2^, Juliana Gadelha Fedeli^2^, Nilo José Coelho Duarte^3^, Léonard De Vinci Kanda Kupa^4^, Victor Matsuno^1^, Claudia Messiano^4^, Silvia Regina Cavani Jorge Santos^4^, David de Souza Gomez^2^

##### ^1^ICr – Institute of Paediatrics, HCFMUSP, São Paulo, SP, Brazil; ^2^DCPQ – Division of Plastic Surgery and Burns, ICHC-FMUSP, São Paulo, SP, Brazil; ^3^DLC – Division of Central Laboratory, HCFMUSP, São Paulo, SP, Brazil; ^4^PC – Pharmacokinetics Center, FCF USP, São Paulo, SP, Brazil

**Background**

A vancomycin initial dose regimen is recommended for critically ill pediatric patients with bloodstream infection caused by Gram-positive strains. Drug effectiveness must be guaranteed by the area under the curve/MIC ratio (AUC/MIC) [1,2].

**Objective**

The aim of the study was to evaluate if dose adjustment at the earlier period of septic shock must be performed for target attainment against Gram-positive MIC 1 mg/l strains based on a pharmacokinetics/pharmacodynamics (PK/PD) approach in burn and nonburn patients.

**Methods**

Thirty-four septic pediatric burn and nonburn patients (12 female and 22 male), with median creatinine clearance (CrCl) >240 ml/min, aged 5–10 years and weighing 16–22 kg (quartiles) were included in the study. Therapy started with 40–60 mg/kg daily equiv. 10–15 mg/kg every 6 h, and the dose was adjusted if required based on the PK/PD target (AUC/MIC >400). Blood was sampled (1.5 ml each) at hours 3 and 5 after starting infusion; serum levels were obtained by immunoassay.

**Results**

We showed significant differences between the recommended dose regimen and individualized therapy (Fig. 1A and B). The percentage of target attainment (PTA) is illustrated after both therapies in those with burns and those without in Fig. 1C and D. Changes in pharmacokinetics are described for both groups by comparison with data previously reported in healthy volunteers (Fig. 2) [3]. After dose adjustment, the target was attained up to MIC 1 mg/l strains for all patients. The target was reached in 65% (13/20) of burn patients and in 43% (6/14) of nonburn individuals against MIC 2 mg/l strains.

**Conclusions**

Since vancomycin pharmacokinetics were altered in septic pediatric patients, when nosocomial Gram-positive strains MIC >0.5 mg/l were isolated, the dose must be adjusted to 80 mg/kg daily equiv. 20 mg/kg every 6 h to eradicate *S. aureus* MIC 1–2 mg/l. A vancomycin PK/PD approach performed in real time permits an earlier clinical intervention and desired clinical outcome with a cure of the infection.

**References**

[1] Gomez DS, et al. Individualized vancomycin dose for paediatric burn patients to achieve PK/PD targets. Burns 2013;39(3):445–50.

[2] Santos et al. Badajoz: Formatex Research Center. 2015;2:808-821

[3] Boeckh M, Lode H, Borner K, et al. Pharmacokinetics and serum bactericidal activity of vancomycin alone and in combination with ceftazidime in healthy volunteers. Antimicrob Agents Chemother 1988;32(1):92–5.

**Fig. 1 (Abstract P34).**
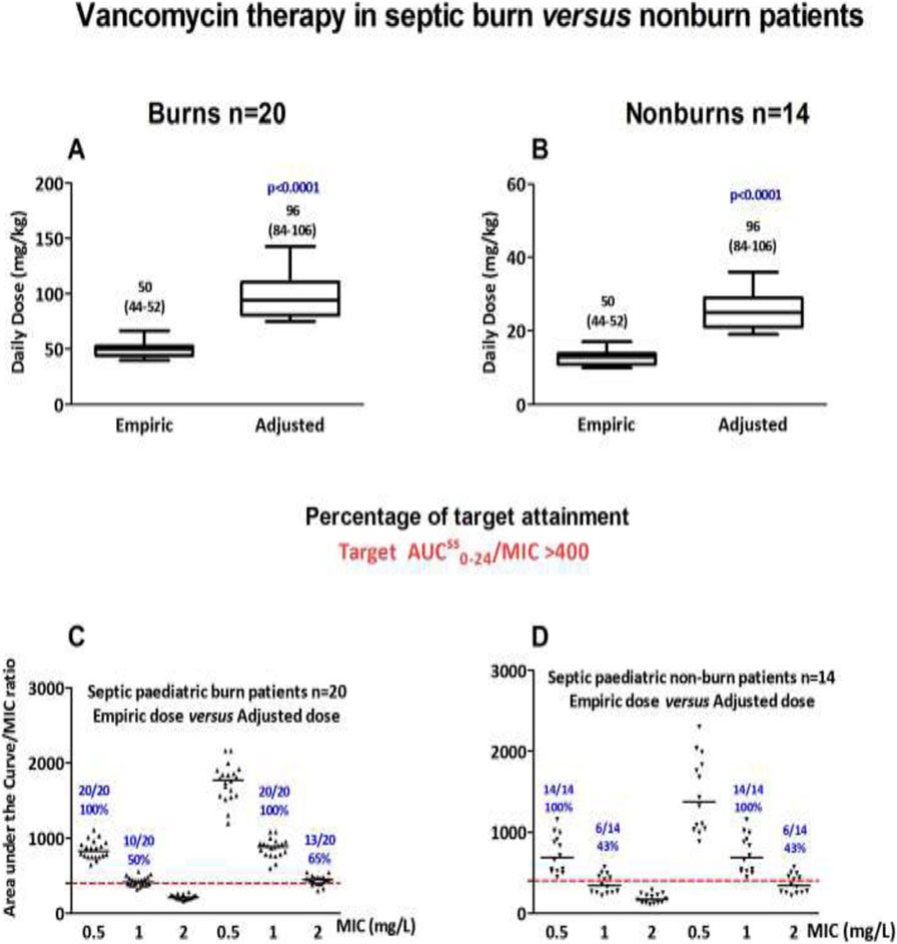
Vancomycin therapy for drug effectiveness in septic pediatric patients. Values are shown as medians (quartiles)

**Fig. 2 (Abstract P34).**
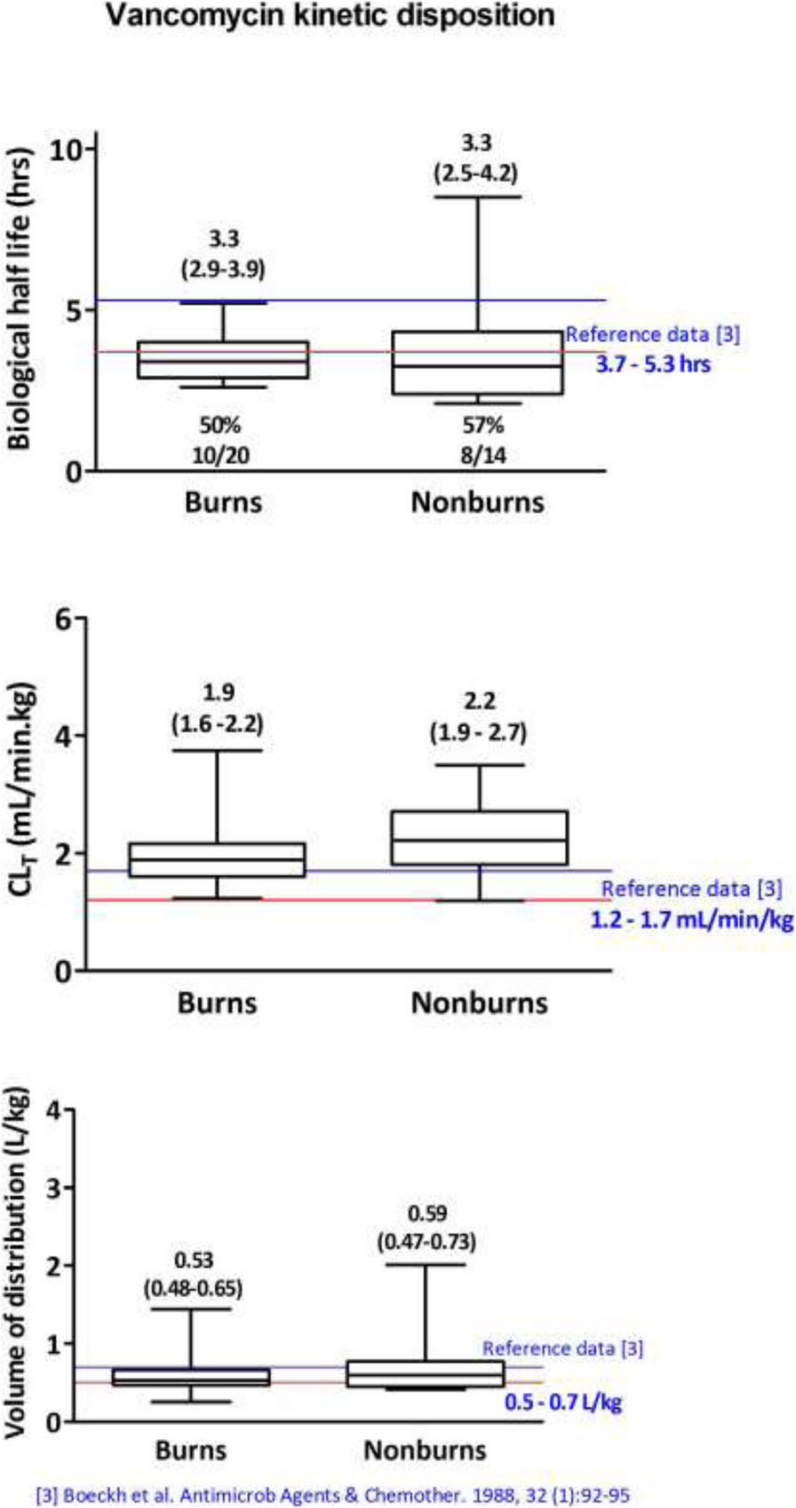
See text for description

### P35 Extended two-hour infusion improves piperacillin effectiveness against susceptible strains in septic burn patients

#### Vanessa Kasubeck de Souza^1^, Elson Mendes Silva Junior^2^, Victor Matsuno^2^, Aline Sandre Gomides^2^, João Manoel da silva Junior^2^, Thiago Camara Oliveira^2^, Gabriela Aparecida Ferreira^2^, David de Souza Gomes^2^, Silvia Regina Cavani Jorge Santos^1^

##### ^1^Pharmacokinetics Center, University of Sao Paulo, São Paulo, SP, Brazil; ^2^Division of Plastic Surgery and Burns, HC FMUSP, São Paulo, SP, Brazil

**Background**

Piperacillin/tazobactam is largely prescribed in the intensive care burn unit (ICBU) for patients with infections caused by Gram-negative strains. The recommended dose cannot achieve the target once serum levels are below those required for effectiveness [1].

**Objective**

The aim of the study was to investigate if the empiric dose of 4.5 every 6 h by 2-h infusion can provide drug effectiveness by a pharmacokinetics/pharmacodynamics (PK/PD) approach in septic burn patients against susceptible MIC 8 mg/l strains.

**Methods**

Ethical approval was obtained from the hospital Ethics Committee (no. 069/09-2015). We studies nine burn patients (three female and six male) with preserved renal function undergoing treatment for septic shock; they had a median age of 32 years, weight of 72 kg, and 29% total burn surface area. Mechanical ventilation plus vasopressors were used in 8/9 patients, and inhalation injury was present in 6/9 of them. Only two blood samples were collected (1.5 ml each) for drug serum measurement by liquid chromatography. Pharmacokinetics (PK) data obtained from patients were compared with results from healthy volunteers [2]. A PK/PD approach was performed to estimate the probability of target attainment (PTA) based on the new predictive index of drug effectiveness (100% fT > MIC) [3].

**Results**

A piperacillin serum trough after a 2-h infusion is illustrated in Fig. 1A and B. Target was attained for all patients by eradication of Gram-negative strains *Pseudomonas aeruginosa* and *Enterobacteriaceae* against MIC 8 mg/l (Fig. 1C). Changes in pharmacokinetics occurred as shown in Fig. 2.

**Conclusions**

Piperacillin pharmacokinetics are altered in septic burn patients improving drug effectiveness. A clinical cure occurred in all patients against isolated susceptible strains after the recommended dose was administered by extended infusion.

**References**

[1] Silva Junior et al. Crit Care 2017;21 (Suppl 2):31.

[2] Occhipinti DJ, Pendland SL, Schoonover LL, et al. Pharmacokinetics and pharmacodynamics of two multiple-dose piperacillin-tazobactam regimens. Antimicrob Agents Chemother 1997;41:2511–7.

[3] Calier M, Stove V, Wallis SC, et al. Assays for therapeutic drug monitoring of β-lactam antibiotics: a structured review. Int J Antimicrob Agents 2015;46:367–75.

**Fig. 1 (Abstract P35).**
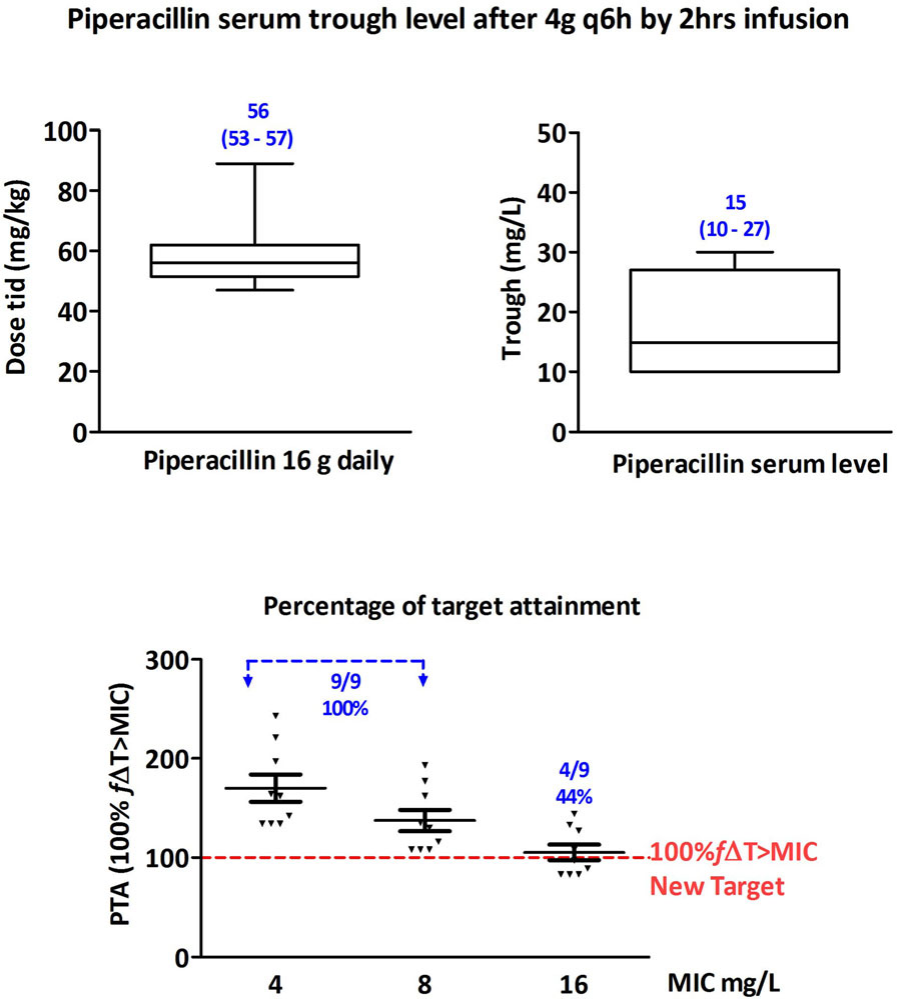
(A, B) Piperacillin therapy in septic burn patients. Values are shown as medians (quartiles). (C) Target attainment in septic burn patients. Values are shown as medians

**Fig. 2 (Abstract P35).**
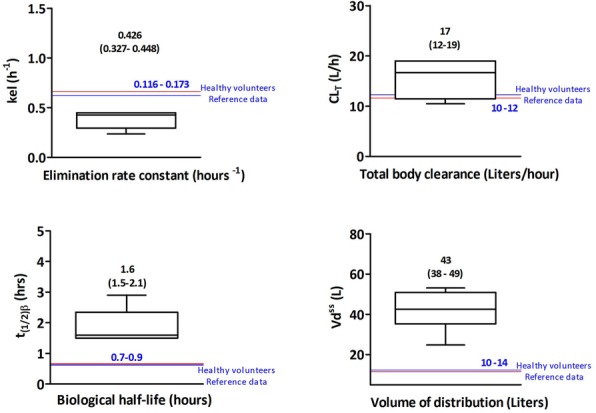
Piperacillin kinetic disposition

### P36 Accuracy of troponin I as a marker in the differentiation of primary or secondary infarction due to sepsis: the RESCA and SAPIENS Registries

#### Michel Pordeus Ribeiro^2^, Pedro Henrique Correia Filgueiras^1^, Rogério da Hora Passos^2^, Paula Chaves Santana Ribeiro^2^, Marcio Oliveira Silva^2^, Paulo Benigno Pena Batista^2^, Luis Claudio Lemos Correia^1,2^

##### ^1^EBMSP – Escola Bahiana de Medicina e Saúde Pública, Salvador, BA, Brazil; ^2^HSR – Hospital São Rafael, Salvador, BA, Brazil

**Background**

Serum troponin is a specific marker for myocardial necrosis. However, it is not specific for a coronary etiology (primary infarction) because it may change in systemic situations of reduced circulatory supply or increased demand. In these situations of instability, physicians may judge significant increases in troponin as a primary infarction, triggering inappropriate invasive coronary procedures.

**Objective**

We aimed to assess the accuracy of a troponin elevation magnitude in differentiating primary infarction or sepsis in critically ill patients who were troponin positive.

**Methods**

From the Acute Coronary Syndrome Registry (beginning in 2010) and Sepsis Registry (beginning in 2013) in our hospital, patients with non-ST elevation myocardial infarction and sepsis, respectively, were consecutively selected, both groups consisting of individuals admitted to intensive care and whose troponin I was positive (detection ≥0.012 μg/l; VITROS, Johnson & Johnson). Patients with creatinine on admission ≥1.5 mg/dL were excluded. Peak troponin values were tested for the differentiation of these two groups.

**Results**

A total of 337 patients with infarction (age 64 ± 13 years, 60% men, 92% in Killip I, 0% Killip IV) were compared with 41 patients with sepsis (age 69 ± 16 years, 59% men, systolic blood pressure 121 ± 33 mmHg, 27% admitted with vasoactive drugs). The median peak of troponin I in infarction was 0.33 μg/l (interquartile range (IQR) 0.06–3.2), a value three times higher than the median found in septic patients (0.11 μg/l, IQR 0.02–0.37). A 10-fold increase over the 99th percentile value was prevalent in both groups and more frequent in the infarction group (50% versus 29%; *p* = 0.01). The 90th percentile of the peak troponin I was 15 μg/l in the infarction group and 7.8 μg/l in the sepsis group. The area under the receiver operating characteristic curve for troponin of 0.65 was nonsignificant in differentiating the two conditions (95% confidence interval 0.56–0.74; *p* = 0,002).

**Conclusions**

Patients with non-ST elevation myocardial infarction present higher troponin elevations when compared with septic patients. However, the accuracy of troponin I to differentiate the two conditions is questionable and a significant proportion of septic patients present with substantial elevations in this marker.

### P37 Evolution of cases of sepsis in adult burn patients

#### Alan Neiverth^1^, Lucas Rodrigues Prim^1^, Renato Nisihara^1^, Cláudio Luciano Franck^2^

##### ^1^FEMPAR – Faculdade Evangélica Mackenzie do Paraná, Curitiba, Pr, Brazil; ^2^HUEM – Hospital Universitário Evangélico Mackenzie, Curitiba, PR, Brazil

**Background**

Sepsis is currently the leading cause of death in burn patients. It is possible that the incidence of infection, increased total body surface area (TBSA), and inhalation injury require a longer hospital stay. There are few studies on sepsis in burn patients on the intensive care unit (ICU).

**Objective**

We aimed to determine the demographic profile, clinical presentation, evolution, procedures, and treatments used in burn patients affected by sepsis attending a university hospital.

**Methods**

This retrospective study was performed in a university hospital ICU by analyzing medical records of adult burn patients who developed sepsis from November 2015 to May 2018. We analyzed patient information, internment, and sepsis. The Sepsis-3 criteria were used for confirmation of a sepsis diagnosis.

**Results**

We studied 44 patients with an average age of 42.1 ± 16.88 years, with 75% of them being male. The median TBSA burned was 50% and was significantly associated with mortality (*p* = 0.013). Mortality was 50%. The median length of stay at hospital was 35 days and on the ICU was 21.5 days. Orotracheal intubation and tracheostomy were the most prevalent aggravations during the internment, occurring in 77.2% and 56.8% of patients, respectively. The median time to first sepsis episode was 7 days and the average total time in sepsis was 13.2 days. The median length of hospital stay in patients with septic shock who died was significantly lower than those who lived (*p* = 0.031). Bacterial culture was positive in 79.5% of cases and the majority of these were typical ICU bacteria. Broad-spectrum antibiotics were administered, the most used being meropenem and vancomycin.

**Conclusions**

This study shows that sepsis occurs more frequently in men, with higher TBSA burned, large internment time, and with aggravating complications. The infections were caused by typical ICU bacteria causing patient mortality of 50%, mostly by septic shock.

## Surgery/Trauma

### P38 Measuring patient dependency in an emergency room: evaluation of patients with severe trauma

#### Genesis Barbosa^1,2^, Regina Sousa^1^

##### ^1^USP – Universidade de São Paulo, São Paulo, SP, Brazil; ^2^UFRJ – Universidade Federal do Rio de Janeiro, Macaé, RJ, Brazil

**Background**

The emergency department is a dynamic environment in which a large volume of patients with different levels of urgency should be evaluated and treated in a timely manner, especially by the nurses who provide first care. Patients with trauma may have different degrees of severity related to the mechanism of trauma (blunt, penetrating, or both) and each level of severity may determine more or less dependency on care.

**Objective**

The aim of this study was to measure blunt and penetrating trauma patient dependency during a stay in the emergency room (ER).

**Methods**

A cross-sectional study was carried out on patients with severe trauma who were assisted in an ER in the city of Rio de Janeiro. Adult patients who died with blunt or penetrating trauma were evaluated for their dependency level during the first-aid stay using the Brazilian version of the Jones Dependency Tool (JDT) [1]. Data were collected in May and June 2018. To estimate the severity of trauma, the Revised Trauma Score (RTS), Injury Severity Score (ISS), New Injury Severity Score (NISS), and Trauma and Injury Severity Score (TRISS) indices were used. The severity was compared according to the type of trauma. The Cronbach's alpha was used to verify the reliability of the JDT.

**Results**

There was predominance of males (83.3%), blunt trauma (66.7%), and red screening category (79.2%). As for the mechanism of trauma, falls and penetrating injuries (firearms and knives) accounted for 33%. The mean age was higher among patients with blunt trauma (50.5 ± 23.6 years). There was an equivalence in the severity of trauma when the mean RTS, ISS, and NISS indices were analyzed. Mean TRISS was higher in patients with penetrating trauma (85.1 ± 24.8) (Table 1). The reliability of the JDT was 0.91. In patients with blunt trauma, there was a predominance of high and moderate dependency of 40%. In patients with penetrating trauma, only high and total dependency levels were observed of 50% (Fig. 1).

**Conclusions**

In patients with blunt and penetrating trauma, dependency levels represented the demand for care required by patients in the ER. There was an indication of greater dependency in patients with greater trauma severity. The JDT proved to be valid and sensitive in the measurement of patient states.

**Reference**

[1] Andrade K, Okuno M, Campanharo C, Batista RE. Tradução e adaptação transcultural do “Jones Dependency Tool” para o português brasileiro. REE 2014;16(4):754–8.

**Table 1 (Abstract P38).** Age, RTS, ISS, NISS and TRISS in patients with blunt and penetrating trauma

	Blunt trauma			Penetrating trauma		
	Mean ± SD	Minimum	Maximum	Mean ± SD	Minimum	Maximum
Age (years)	50.5 ± 23.6	18	95	45.2 ± 21.2	29	95
RTS	5.4 ± 1.4	2.8	7.8	6.0 ± 1.8	1.8	7.8
ISS	15.8 ± 4.9	10	29	14.1 ± 3.0	9	17
NISS	28.3 ± 11.3	11	43	29 ± 7.6	17	43
TRISS	75.1 ± 28.8	2.6	98.4	85.1 ± 24.8	20.1	98.8

*ISS* Injury Severity Score, *NISS* New Injury Severity Score, *RTS* Revised Trauma Score, *SD* standard deviation, *TRISS* Trauma and Injury Severity Score

**Fig. 1(Abstract P38).**
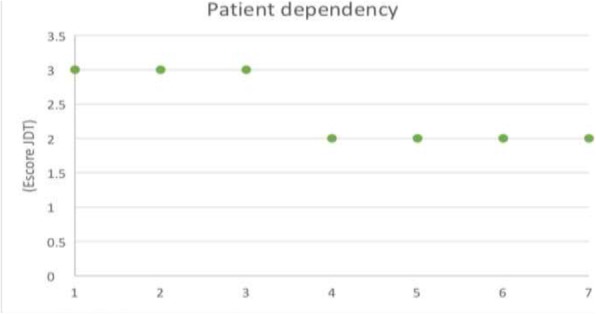
Patient dependency in the emergency room stay. JDT Jones Dependency Tool

### P39 Mortality trends in high-risk surgical patients: a comparative analysis over 10 years of evolution in two Brazilian multicenter cohorts

#### João M Silva Jr^1,2^, Renato CF Chaves^1^, Thiago D Corrêa^1^, Murillo S Assunção^1^, Cristina P Amendola^3^, Ary Serpa-Neto^1^, Henrique T Katayama^2^, Luiz MS Malbouisson^2^, Suzana M Lobo^4^

##### ^1^HIAE – Hospital Israelita Albert Einstein, São Paulo, SP, Brazil; ^2^ICHCFMUSP – Divisão de anestesiologia, Hospital das Clínicas, FMUSP, São Paulo, SP, Brazil; ^3^HCB – Fundação Pio XII, Hospital de Câncer de Barretos, Barretos, SP, Brazil; ^4^FAMERP – Faculdade de Medicina de São José do Rio Preto, Hos, São José do Rio Preto, SP, Brazil

**Background**

Many advances in the care of high-risk surgical patients have been made. Nevertheless, temporal trends in outcomes in this population of patients in developing countries are lacking.

**Objective**

This study aimed to investigate trends in mortality and complications in high-risk surgical patients over a 10-year period.

**Methods**

Data from two prospective multicenter cohort studies (SCORIS and BRASIS studies) conducted by the AMiB Network from 2008 to 2018 to address the outcomes of high-risk surgical patients in Brazil were compared. All noncardiac surgical patients admitted to intensive care units (ICUs) were daily followed-up to hospital discharge to determine complications and mortality.

**Results**

Individual patient data from 1491 high-risk surgical patients (904 patients from the BRASIS study and 587 from the SCORIS study) in 50 ICUs were enrolled in this analysis. The mean age was 61.0 ± 17.4 years, 54.2% were male, and 47.8% had ASA 2 physical status followed by 34.9% with ASA 3 status. The crude hospital mortality rate decreased from 20.6% (95% confidence interval (CI) 17.1–24.6%) in 2008 to 8.9% (95% CI 7.0–11.2%) in 2018. After adjusting for baseline Sequential Organ Failure Assessment (SOFA) score, age and type of ICU, patients enrolled in 2018 (10.1%, 95% CI 7.5–13.4) compared with those enrolled from 2008 (16.1%, 95% CI 12.8–20.0) exhibited a lower risk of hospital death (odds ratio (OR) 0.58, 95% CI 0.40–0.85; *p* = 0.005) and ICU mortality rate (5.5% in 2018 and 11.2% in 2008; OR 0.46, 95% CI 0.29–0.75; *p* = 0.001). The frequency of infection decreased significantly from 19.8% in 2008 to 2.9% in 2018 (OR 0.12,; 95% CI 0.07–0.21; *p* < 0.001) while the general rate of complications did not differ between the two periods, 34.8% (95% CI 29.8–40.3%) in 2008 and 33.8% (95% CI 28.9–39.2%) in 2018.

**Conclusions**

Over the 10-year period between 2008 and 2018, mortality and incidence of infection improved in high-risk surgical patients admitted to Brazilian ICUs; however, general complication rates remained unchanged.

**Acknowledgements**

The authors thank the BRASIS study group, Israelita Albert Einstein Hospital and Barretos Cancer Hospital for their support of the study. The study was endorsed by the Brazilian Network of Research in Intensive Care (AMIBnet).

**Ethics Approval**

This study was approved by the research ethics commission at the study coordinating center (CAAE: 55828016.1.1001.0071) and at all participating centers.

**Reference**

[1] Lobo SM, Rezende E, Knibel MF, Silva NB, Paramo JA, Nacul F, et al. Epidemiology and outcomes of non-cardiac surgical patients in Brazilian intensive care units. Rev Bras Ter Intensiva 2008;20(4):376–84.

### P40 Predictors of mortality on admission to the intensive care unit in surgical patients: a 2-year cohort study

#### Fábio Ferreira Amorim^1,2^, Gabriel Kanhouche^1^, Alessandra Vasconcelos da Silva Paiva^2^, André Jaccoud de Oliveira^2^, Carlos Darwin Gomes da Silveira^1,2^, Edmilson Bastos de Moura^1,2^, Flávio Ferreira Pontes Amorim^3^, Marcelo de Oliveira Maia^1^

##### ^1^HSL – Hospital Santa Luzia, Rede D'Or São Luiz, Brasília, DF, Brazil; ^2^ESCS – Escola Superior de Ciências da Saúde, Brasília, DF, Brazil; ^3^UCB – Universidade Católica de Brasília, Brasília, DF, Brazil

**Background**

Patients who undergo high-risk surgical procedures represent a large proportion of intensive care unit (ICU) admissions. The accurate identification of patients who are at high risk of complications and death on ICU admission is important for the management of these patients [1–3].

**Objective**

The aim of this study was to assess the prognostic factors in the first 24 h of admission for ICU mortality in a surgical ICU.

**Methods**

This was a prospective cohort study conducted on patients admitted to the surgery ICU of the Hospital Santa Luzia Rede D'Or, Brasília, DF, Brazil, over 2 years. Patients transferred to another ICU were excluded.

**Results**

Of 1185 patients included, the mean age was 57 ± 17 years, the Acute Physiology and Chronic Health Evaluation (APACHE) II score was 8 ± 5, the Simplified Acute Physiology II score (SAPS 2) was 23 ± 12, and 91% underwent elective surgery. ICU mortality was 3.5%. The nonsurvivors were older (68±20 vs 56±16, p<0.01), APACHE II (17±9 vs. 7±5, p<0.01), SAPS 2 (42±19 vs. 22±11, p<0.01), SOFA (4±3 vs. 2±1, p<0.01), and arterial lactate level at ICU admission (2.2±2.1 vs 1.4±1.1 mg/dL, p<0.01). There was no difference between groups regarding PaCO2 gap (9.9±5.2 vs. 8.0±4.7, p=0.07), hiponatremia (3.4% vs 3.6%, p=0,93), and SvO2 (75±17% vs. 74±13%, p=0,08). Using multivariate analysis, SOFA (p=0.02), age (p=0.02) and arterial lactate level at ICU admission (p=0.04) were independently associated with ICU mortality.

**Conclusions**

Sequential Organ Failure Assessment (SOFA) score, age, and arterial lactate level at ICU admission were independently associated with the ICU mortality in surgical patients.

**References**

[1] Sobol JB, Wunsh H. Triage of high-risk surgical patients for intensive care. Crit Care 2011;15:217.

[2] Sudarshan M, Feldman LS, St Louis E, et al. Predictors of mortality and morbidity for acute care surgery patients. J Surg Res 2015;193(2):868–73.

[3] Santana-Cabrera L, Martín-Santana JD, Lorenzo-Torrent R, et al. Prognosis of critical surgical patients depending on the duration of stay in the ICU. Int J Crit Illn Inj Sci 2015;5(3):144–148.

### P41 Status of mobility in the intensive care unit: clinical versus surgical patients

#### Andressa Brossi de Figueiredo, Flavia Fernandes Manfredi de Freitas, Ricardo Kenji Nawa, Karina Tavares Timenetsky

##### HIAE – Hospital Israelita Albert Einstein, São Paulo, SP, Brazil

**Background**

Early mobilization is a fundamental aspect of the recovery of critical patients, both clinical and surgical [1], and the functional evaluation using a metric instrument is of great importance.

**Objective**

The objective of this study was to compare mobility between surgical and clinical patients at intensive care unit (ICU) admission versus discharge. The secondary objective was to stratify the evolution of mobility barriers between admission and discharge from the ICU.

**Methods**

This was a retrospective study using a physiotherapy database from the Department of Critical Care of a private hospital in São Paulo, from July 2017 to January 2018. The mobility was evaluated through the Perme Intensive Care Unit Mobility Score instrument at admission and discharge from the ICU [2]. Patients were older than 18 years and hospitalized for at least 24 h in intensive care; patients with previous functional dependence, those who died during intensive care hospitalization, or those with Perme Score instrument filled out incorrectly were excluded. The study was approved by the hospital’s Ethics Committee. There was no consent form as data were extracted from a database where patients were not identified, making it impossible to obtain consent.

**Results**

The study included 628 patients, the majority of whom were males (*n* = 337, 53.6%), with a mean age of 70 ± 17.8 years, and were mostly clinical patients (*n* = 464, 73.8%). The main barriers were mental status, the use of invasive and noninvasive mechanical ventilation, and pain. There was an improvement in mobility at discharge from intensive care compared with admission (17.3 ± 8.8 versus 9.1 ± 7.2, *p* (17.3 ± 8.8 x 9.1 ± 7.2, p <0.001). Surgical patients had a higher mobility than the clinical patients (19 ± 8.3 x 16.7 ± 8.9; p = 0.001) on discharge day (Figure 1). All barriers decreased until discharge from the intensive care unit. Factors associated with improved mobility: surgical patients, decreased level of consciousness and no ventilatory support at admission.

**Conclusions**

Patients presented improvement in their mobility status upon discharge from intensive care compared with admission; surgical patients had higher levels of mobility than patients, and all items considered as barriers decreased during hospitalization.

**References**

[1] Hodgson CL, Berney S, Harrold M, Saxena M, Bellomo R. Clinical review: early patient mobilization in the ICU. Crit Care 2013;17(1):207.

[2] Perme C, Nawa RK, Winkelman C, Masud F. A tool to assess mobility status in critically ill patients: the Perme Intensive Care Unit Mobility Score. Methodist DeBakey Cardiovascular Journal 2014;10(1):41.

**Fig. 1 (Abstract P41).**
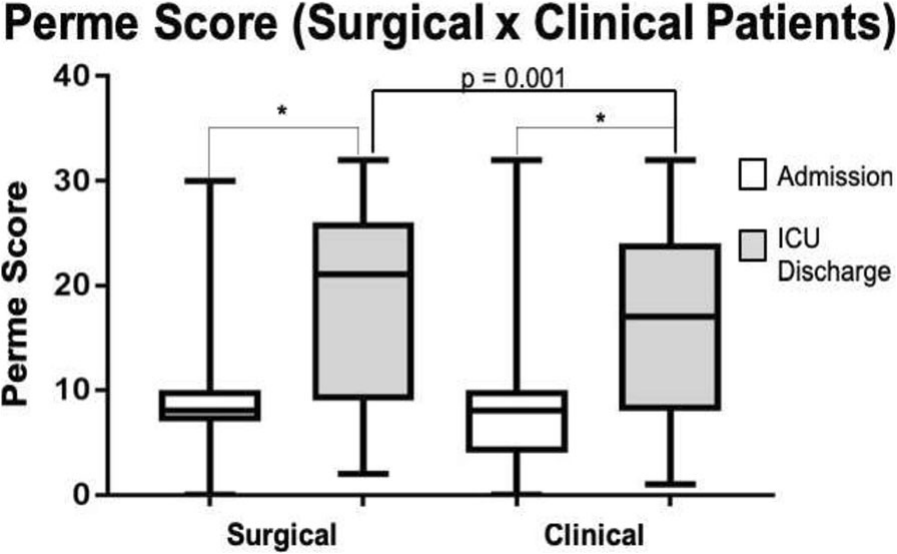
Perme score of surgical and clinical patients at intensive care unit (ICU) admission and discharge (* p <0.05)

